# Separation and flow cytometry analysis of microplastics and nanoplastics

**DOI:** 10.3389/fchem.2023.1201734

**Published:** 2023-09-15

**Authors:** Jingjing Li, Fuyi Huang, Guohui Zhang, Zixing Zhang, Xian Zhang

**Affiliations:** ^1^ Key Laboratory of Urban Environment and Health, Institute of Urban Environment, Chinese Academy of Sciences, Xiamen, China; ^2^ University of Chinese Academy of Sciences, Beijing, China; ^3^ College of Life Sciences, Fujian Agriculture and Forestry University, Fuzhou, China

**Keywords:** counting region, flow cytometry, microplastics, multistage filtration, nanoplastics, Nile Red

## Abstract

In recent years, the utilization of flow cytometry for quantitative microplastic analysis has gained prominence. However, the current methods have some drawbacks that need to be improved. The present study aims to enhance the flow cytometry detection protocols for Nile red (NR) stained microplastics, facilitating distinct microplastic and nanoplastic enumeration. By elevating dimethyl sulfoxide (DMSO) concentration to 20%–30% within the solution, NR solubility improved and agglomeration reduced. The analysis of 26 replicates of polystyrene (PS) liquid samples through four distinct dot plots highlighted the superior accuracy of dot plots integrating yellow fluorescence. Through systematic staining of varying NR concentrations across three microplastic liquid samples (polyethylene terephthalate, polyethylene, and polypropylene), the optimal staining concentration was determined to be 15–20 μg/mL. The distributions of agglomerated NR and NR stained PS under two scenarios—dissolved NR and partially agglomerated NR—were compared. Results showed their distinct distributions within the side scatter versus yellow fluorescence dot plot. Counting results from gradient-diluted PS liquid samples revealed a microplastic detection lower limit of 10^4^ particles/mL, with an optimal concentration range of 10^5^–10^6^ particles/mL. Flow cytometric assessment of PS microspheres spanning 150 nm to 40 μm indicated a 150 nm particle size detection minimum. Our investigation validated the efficacy of NR staining and subsequent flow cytometry analysis across eleven types of microplastics. Separation and concentration of microplastics (1.0–50.0 μm) and nanoplastics (0.2–1.0 μm) were achieved via sequential sieving through 50, 1.0, and 0.2 μm filter membranes. We used a combination of multiple filtration steps and flow cytometry to analyze microplastics and nanoplastics in nine simulated water samples. Our results showed that the combined amount of microplastics (1.0–50.0 μm) and nanoplastics (0.2–1.0 μm) after filtration had a ratio of 0.80–1.19 compared to the total microplastic concentration before filtration. This result confirms the practicality of our approach. By enhancing flow cytometry-based microplastic and nanoplastic detection protocols, our study provides pivotal technical support for research concerning quantitative toxicity assessment of microplastic and nanoplastic pollution.

## 1 Introduction

According to the particle size, microplastic particles can be divided into microplastics and nanoplastics. Microplastics generally refer to plastic particles having a size range of 1 μm–5 mm (Lv et al., 2019), while nanoplastics are defined as plastic particles with a size of less than 1,000 nm (Gigault et al., 2018). Common microplastic particles mainly include polyethylene (PE), polypropylene (PP), polyvinyl chloride (PVC), polystyrene (PS), and polyethylene terephthalate (PET) (Adhikari et al., 2022). Microplastic particles are present ubiquitously in rivers, lakes, oceans, air, soil, drinking water, and various environmental ecosystems (Alimba and Faggio, 2019). Due to their wide distribution, small particle size, and adsorbability of toxic pollutants, microplastics have been listed as one of the top ten emerging important pollutants by the United Nations Environment Programme (UNEP) in 2014 (Kiran et al., 2022).

Microplastics and nanoplastics can be ingested by microorganisms and animals in water, soil, and sediment and enter the food chain, causing toxic effects on organisms (Yong et al., 2020). The particle size has significant effects on the biological toxicity of microplastic particles, and the smaller the particle size the greater the risk of ingestion by organisms (Bhagat et al., 2020; Banerjee and Shelver, 2021). Microplastic particles less than 1 μm have a higher risk of accumulation and transfer in different tissues and organs of organisms (Xu et al., 2020). In recent years, people have gradually realized that microplastic particles also cause serious harm to human health. Microplastics and nanoplastics can enter the human body through skin absorption, air exposure, drinking water, food with plastic containers, personal care products, etc. (Revel et al., 2018), while they can stay in various tissues and organs of the human body with blood circulation (Hirt and Body-Malapel, 2020; Yee et al., 2021). Some published studies confirmed that microplastics and nanoplastics exist in the blood, placenta, and cirrhotic liver tissue of humans (Ragusa et al., 2021; Horvatits et al., 2022; Leslie et al., 2022). The harm of microplastic particles to the human body includes carcinogenic risk, reproductive toxicity, developmental toxicity, neurotoxicity, etc (Lehner et al., 2019; Yee et al., 2021). Therefore, it is of great significance to conduct in-depth research in this field.

Evaluating the biological toxicity of microplastic particles gives the urgent need for quantitative analysis of microplastics and nanoplastics using an appropriate method. Currently, the most commonly utilized methods for microplastic counting are Raman spectroscopy and microscopy (Jin et al., 2022). Infrared microscopy is suitable exclusively for plastic particles exceeding 20 μm in size (Huang et al., 2022), while scanning electron microscopy is effective for detecting microplastics with a minimum particle size of 1 μm (Adhikari et al., 2022). Additionally, the detectable limit size for plastic particles using Raman spectroscopy is 1 μm (Kundu et al., 2021). Nevertheless, it is important to note that all of these methods are restricted to counting microplastics above the micron scale. Flow cytometry can quantitatively analyze the number of cells and microparticles with fluorescent staining (Manohar et al., 2021). Various dyes find application in microplastic staining, with Nile Red (NR), Fluorescein, Rhodamine, Calcofluor White, Gentian Violet, and Toluidine Blue O being among the common choices (Lv et al., 2019). Notably, NR stands out as the predominant fluorescent dye utilized for microplastics (Shim et al., 2016; Shruti et al., 2022). In recent years, some researchers have attempted to quantitatively analyze microplastics using flow cytometry (Kaile et al., 2020; Tse et al., 2022; Bianco et al., 2023). Microplastics stained with NR in the solutions, including 10% dimethyl sulfoxide (DMSO) (Kaile et al., 2020), 0.1% Tween 20 (Tse et al., 2022), or ultrapure water (Bianco et al., 2023) were counted by flow cytometry. However, these existing methods have some shortcomings. NR is a lipophilic oxazine fluorescent dye with strong hydrophobicity, which is prone to aggregate and precipitate in water. It is difficult to distinguish stained plastic particles from aggregated NR in the dot plot since they are distributed in the same region (Kaile et al., 2020). In addition, microplastics and nanoplastics cannot be counted respectively by flow cytometry without a sorting function. It is significant to separate microplastics and nanoplastics and count them respectively.

This study is aimed to analyze plastic particles with a size of less than 50 μm by flow cytometry. The two purposes of this study are: 1) to improve the NR staining conditions for plastic particles to reduce the noise of agglomerated NR, and 2) to separate and quantify microplastics and nanoplastics. We improved the NR staining protocols by optimizing staining conditions and selecting an appropriate counting region. Microplastic liquid samples were sieved through 50, 1.0, and 0.2 μm filter membranes orderly to separate microplastics with the size range of 1.0–50.0 μm, 0.2–1.0, and 0–0.2 μm. Through multistage filtration, NR staining, and flow cytometry, the microplastics (1.0–50.0 μm) and nanoplastics (0.2–1.0 μm) were counted respectively. This study improves the detection protocols of microplastics and nanoplastics by flow cytometry, which is significant for the evaluation of the toxicity of microplastics and nanoplastics.

## 2 Materials and methods

### 2.1 Microplastic particles

There were eleven different types of microplastic used in this study, including polyvinylidene fluoride (PVDF), polyvinylidene chloride (PVDC), polyethylene terephthalate (PET), polyethylene (PE), polytetrafluoroethylene (PTFE), ethylene-chlorotrifluoroethylene (ECTFE), polymethyl methacrylate (PMMA), polyvinyl chloride (PVC), polyfluoroalkoxy (PFA) and polystyrene (PS). Five different sizes of PS microspheres (40 μm, 10 μm, 1 μm, 400 nm, and 150 nm) were purchased as standard microplastics and nanoplastics. All these microplastics and nanoplastics were suspended in ethanol (analytical reagent, AR) to prepare the microplastic stocks. The brand and size information of microplastics and nanoplastics used in this study is shown in Supplementary Table S1.

### 2.2 Optimization of NR staining protocols

10 mg of NR dye (RHAWN, China) was dissolved in 10 mL of dimethyl sulfoxide (DMSO, AR) to prepare the NR stock solution, with a final concentration of 1 mg/mL. The NR stock solution was sieved through a 50 μm stainless steel mesh after an ultrasonic treatment for 10 min. Each 100 μL of NR stock solution was divided into 2 mL brown glass vials and stored at 4°C. Before each staining, the melting NR stock solution was conducted with ultrasonic treatment for 10 min and then sieved through a 0.22 μm filter with a 13 mm diameter, to remove the precipitated and aggregated NR. We conducted a comparison of the removal effect on NR precipitation by three different filters, including a 0.22 μm glass fiber (GF) filter (BKMAM, China), a 0.22 μm mixed cellulose esters (MCE) filter (BKMAM, China), and a 0.22 μm nylon filter (JinTeng, China).

This study investigated solvents and solutions to reduce the aggregation of NR. In this study, four solvents were selected as NR solvents, including DMSO (AR), ethanol (AR), acetone (AR), and acetonitrile (AR). The solubility of NR in 0.1% Tween 20% and 10% DMSO was compared in this study. We investigated the effect of increasing the ratio of DMSO in solution on the solubility of NR. We also confirmed the optimum NR final concentration and the optimum particle concentration of plastic particles detected by flow cytometry. All the optimum conditions were determined by analyzing the population distribution of NR and stained PS in the dot plot (side scatter versus yellow fluorescence) under different conditions.

### 2.3 Scanning laser confocal microscope

100 μL of PS standard microsphere (150 nm, 400 nm, 1 μm, 10 μm, and 40 μm) stocks were diluted with 50% DMSO to prepare the PS microsphere liquid samples. 100 μL of PS microspheres liquid samples were added into a 2 mL brown glass vial with 1.5 μL of 1 mg/mL NR stock solution. After the vortex, the PS microspheres were stained for 20 min. Then, 20 μL of the stained PS microspheres liquid samples were transferred on microscope slides (FanYi, China) and placed on the scanning laser confocal microscope (LSM 710, ZEISS, Germany) for imaging analysis.

### 2.4 Flow cytometry analysis

Each liquid sample was sieved through 50 μm stainless steel mesh before NR staining. 100 μL of each sample was added to 1.5 μL of 1 mg/mL NR stock solution in a 1.5 mL centrifuge tube (Thermo Fisher Scientific) or a 2 mL brown glass vial. After vortex mixing, the samples were incubated at room temperature for 10 min. Each sample was vortex-mixed before analysis. Plastic particles and liquid samples were analyzed under the flow cytometer (Guava easyCyte, Luminex) using a blue laser (488 nm excitation). The voltage parameters were set as 140 for forward scatter (FSC), 280 for side scatter (SSC), 100 for green fluorescence, 430 for yellow fluorescence (YEL), and 610 for red fluorescence (RED) (Tse et al., 2022). The unstained plastic liquid samples and NR were analyzed as the negative control. The sequence of loading and analysis is as follows: first, the unstained plastic particle samples, followed by the NR negative control samples, and finally, the stained plastic particle samples. A quick washing was performed with 10%–30% DMSO as the cleaning buffer by the flow cytometer using the capillary cleaning tools between each sample to minimize contamination. The detection was repeated in total three times for each sample. The counting region was defined as the region containing the population of stained plastic particles. To ascertain the optimal counting region, we assessed the accuracy of counting results for 26 replicates of PS microsphere liquid samples and the background noise of 23 replicates of NR negative control. This evaluation involved the analysis of four counting regions derived from four dot plots, including side scatter versus red fluorescence dot plot, side scatter versus yellow fluorescence dot plot, yellow fluorescence versus red fluorescence dot plot, and side scatter versus forward scatter dot plot. These four dot plots correspond to counting regions labeled R14, R15, R16, and R17.

### 2.5 Microscopic observation and counting

The standard plastic particles liquid samples were diluted with 75% ethanol, with an optimum concentration of 10^4^–10^6^ particles/mL. The diluted liquid sample was transferred to the counting chamber of a hemocytometer and then stood for 5–10 min. The hemocytometer was under the microscope (Axio Imager A1, Zeiss, Germany) at ×100 magnification to count the number of plastic particles. Each sample was counted in total three times.

### 2.6 Separation of microplastics and nanoplastics

In this study, a multistage filtration device was designed to separate microplastics and nanoplastics (Supplementary Figure S1). The plastic particles liquid samples were sieved through 50 μm stainless steel meshes, 1.0 μm filter membranes, and 0.2 μm filter membranes. 50 μm stainless steel meshes were used to remove substances with more than 50 μm size. 1.0 μm filter membranes were used to intercept 1.0–50.0 μm plastic particles. 0.2 μm filter membranes were used to intercept 0.2–1.0 μm plastic particles. The size of plastic particles in the filtrate of 0.2 μm filter membranes was 0–0.2 μm. The plastic particles of 1.0 μm and 0.2 μm filter membranes were cut into pieces and suspended with 10%–30% DMSO.

We tested the recovery efficiency of microplastics with three different kinds of filter membranes, including 1.0 μm MCE membranes (Xingya, China), 1.0 μm polycarbonate (PC) membranes (Xingya, China), and 1.0 μm GF filter membrane (GF/B, Whatman). 100 mL of 10–40 μm PS microsphere liquid samples were sieved through three different kinds of filter membranes, respectively. The membranes were collected and cut into pieces, which were suspended in 1 mL of 10%–30% DMSO by brief vortex for 15 s, and then with ultrasonic treatment for 10 min. The elution suspension was sieved through 50 μm stainless steel mesh to remove tiny membrane fragments. After a brief vortex, the PS microspheres elution suspension was counted with a light microscope (Axio Imager A1, Zeiss, Germany).

The elution efficiency of microplastics from MCE membrane was investigated in three different ways, including vortex, ultrasonication, and homogenization. Vortex was performed with a vortex mixer (Vortex-6, Kylin-Bell, China) with an intensity of 8 for 5 min. Ultrasonic treatment was carried out in an ultrasonic washing machine (S10H, Zealway, China) with operating parameters of 100% power for 10 min. Homogenization was carried out on a Homogenizer (Biopre-24, Allsheng, China), and the running parameter was at a speed of 5.5 m/s, 40 s, 1-min intervals, and 2 cycles. The brief operation process is as follows. 10 mL of 10–40 μm PS microsphere liquid samples were sieved through 1.0 μm MCE filter membranes (Xingya, China). The cut membranes were suspended in 1 mL of 10%–30% DMSO, and then eluted by sonication, vortex, or homogenization, respectively. After filtering with 50 μm stainless steel meshes, the PS microsphere elution suspension was counted under a light microscope (Axio Imager A1, Zeiss, Germany).

### 2.7 Preparation of simulated water samples

Due to the low number of microplastic particles in the aquatic ecosystems, it is difficult to filter the water samples to obtain a sufficient particle number of microplastics for flow cytometry. To reduce the difficulty of the experiment, the PS microspheres were added to water samples to prepare the simulated water samples contaminated with microplastics in this study. We collected 3 ultrapure water samples, 3 tap water samples, and 3 lake water samples, which were sieved through 0.1 μm MCE filter membranes (Xingya, China) to remove all particulates. Mixed PS microspheres (400 nm–40 μm) were added to 9 water samples to prepare the simulated water samples, with a final concentration of ∼10^5^ particles/mL. PS microspheres were mixed with 4 different particle sizes of microplastics, including 40 μm, 10 μm, 1 μm, and 400 nm. The ultrapure water without plastic particles was performed as the negative control for simulated water samples. 50 mL of 9 simulated microplastic water samples or ultrapure water were mixed with 50 mL of 30% hydrogen peroxide (H_2_O_2_) and stood overnight (15–18 h) to remove organic carbon. After wet oxidation, 50 mL of simulated environmental samples or ultrapure water samples were treated with multistage filtration to separate microplastics and nanoplastics. The 1.0 μm or 0.2 μm filter membranes were suspended in 1 mL of 30% DMSO and then eluted by ultrasonication. The simulated water samples before filtration, the suspended plastic liquid samples of 1.0 μm filter membranes, and the counterpart of 0.2 μm filter membranes were analyzed using flow cytometry (Guava easyCyte, Luminex). There were 3 replicates for each sample.

The particle concentration of stained microplastics is calculated as the difference between the particle concentration of microplastics stained with NR and the particle concentration of background noise derived from the negative control within the designated counting region. The distinction between NR-stained microplastic samples and the negative control lies in the presence of microplastics in the former and their absence in the latter, while all other components remain identical. In this study, we selected the R15 region in the dot plot of side scatter versus yellow fluorescence as the counting region for microplastic samples. The formula for calculating the particle concentration of microplastics is as follows:

$$\left.C_{NR-MPs}={C\left(R15\right.}_{NR-MPs}\left.\;\right)-{C\left(R15\right.}_{NC}\right)$$

In the above formula, C_NR-MPs_ represents the actual particle concentration of NR-stained microplastics, C(R15_NR-MPs_) is indicative of the particle concentration of the population in the R15 region for NR-stained microplastics samples, and C(R15_NC_) represents the particle concentration of the population in the R15 region for the negative control.

## 3 Results

### 3.1 Optimizing staining protocols to reduce NR precipitation

**FIGURE 1:**
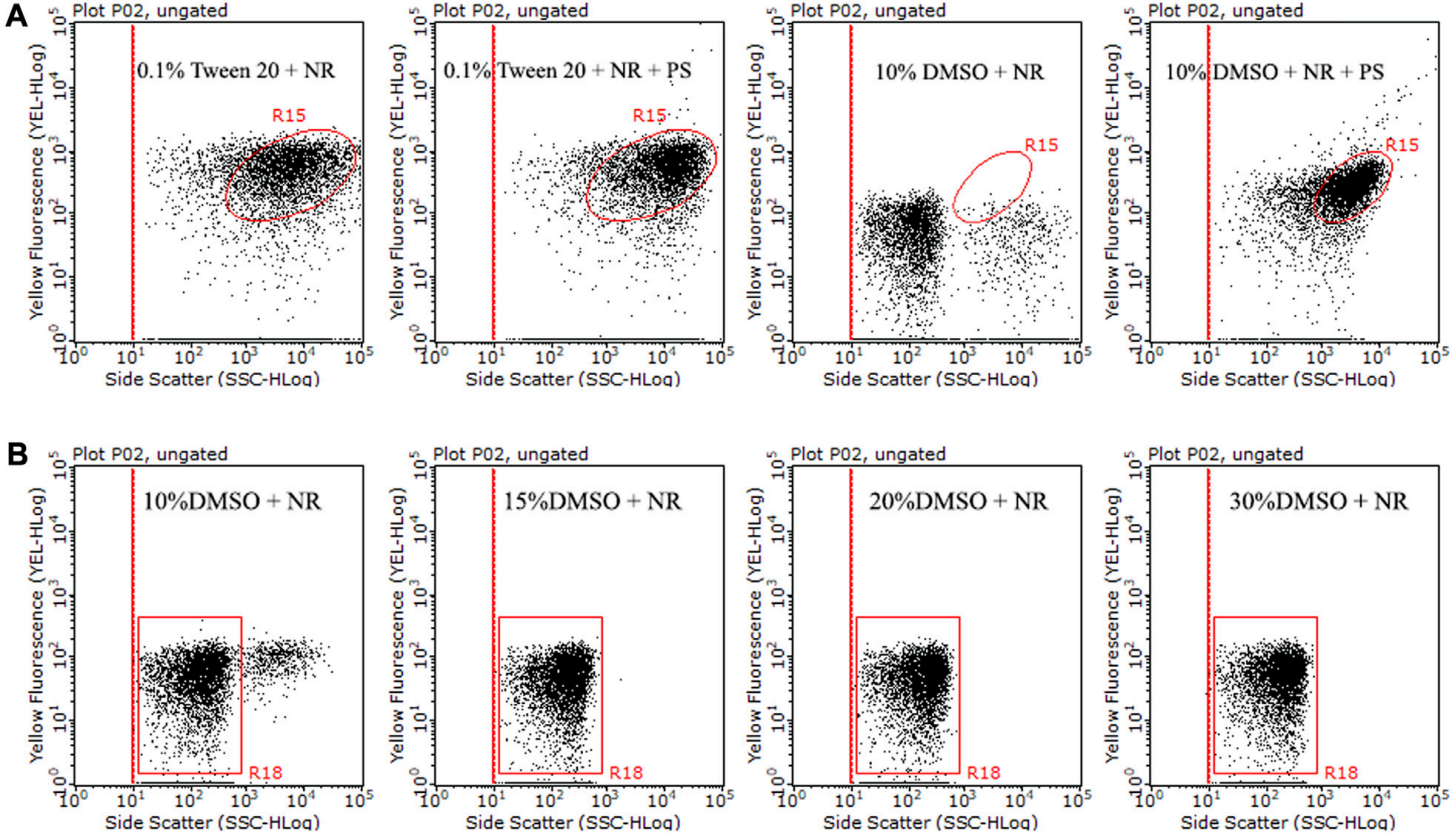
Comparison of side scatter versus yellow fluorescence dot plots of NR and stained PS (10 μm) in 0.1% Tween 20 or 10% DMSO **(A)**. Additionally, a comparison of side scatter versus yellow fluorescence dot plots was conducted for 15 μg/mL NR in 10% DMSO, 15% DMSO, 20% DMSO, and 30% DMSO at room temperature for 10 min **(B)**. The population within the R15 region represents stained microplastics. The R18 region indicates dissolved NR and background noise, while the population situated on the right side of the R18 region symbolizes NR aggregation.

We investigated the effect of the solution on NR solubility. The solubility of NR was compared under two solutions, 0.1% Tween 20% and 10% DMSO (Figure 1A). It was difficult to distinguish the NR population from the stained PS population at the dot plot with 0.1% Tween 20 as the solution (Figure 1A), indicating that NR is easy to aggregate in 0.1% Tween 20. In 10% DMSO, the population of NR was mainly concentrated in the lower left corner of the dot plot, while the counterparts of stained PS were concentrated in the upper right corner of the dot plot (Figure 1A). The results showed that it is easy to distinguish NR from stained PS in the dot plot with 10% DMSO as the solution. The above results indicate that 10% DMSO is more suitable than 0.1% Tween 20 as the solution for NR staining in flow cytometry analysis.

Nevertheless, we found that NR exhibited some agglomeration and precipitation in 10% DMSO, which could lead to false positive results. To improve the solubility of NR and reduce the precipitation of NR in the solution, we increased the ratio of DMSO to 15%–30% in the solution. The dissolution state of NR in 15%–30% DMSO was significantly improved compared with 10% DMSO (Figure 1B). In addition, we compared the dissolution states of different batches of NR stock solution in 15% DMSO, 20% DMSO, and 30% DMSO. Our results showed some batches of NR dissolved well in 15% DMSO, while some batches agglomerated in 15% DMSO (Supplementary Figure S2). Although NR occasionally agglomerated in 20%–30% DMSO, in general, the dissolution state of NR in 20%–30% DMSO had a significant improvement compared with 15% DMSO. In this study, we chose 15%–30% DMSO as the NR staining solution for flow cytometry analysis. However, we moderately adjusted the ratio of DMSO according to the dissolved state of NR.

Solvents are one of the important factors affecting the solubility of NR. In this study, four different solvents, including DMSO, ethanol, acetone, and acetonitrile, were selected as the solvent of NR. We analyzed the dot plots (side scatter versus yellow fluorescence) of NR and stained PS in 15% DMSO when NR stock solution was prepared with four different solvents. The staining efficiency of NR on PS (10 μm) with these four different solvents was 91.98%, 87.02%, 86.85%, and 83.76%, respectively (Supplementary Figure S3). There was some noise in the upper right corner of the NR dot plot when ethanol, acetone, and acetonitrile were used as solvents for NR, while there was minimum noise when DMSO was used as the solvent (Supplementary Figure S3). This result showed that DMSO was a more favorable solvent for NR compared with the other three solvents. Since DMSO has good chemical stability and is also commonly used for flow cytometer analysis, DMSO is finally selected as the solvent for NR in this study.

The population of NR aggregation in the dot plot may originate from the insoluble NR in the NR stock solution. We analyzed the dot plots of the NR stock solution before and after filtration and compared the removal effect of 0.22 μm GF filter, 0.22 μm MCE filter, and 0.22 μm nylon filter on the aggregated NR. Before filtration, two distinct populations were evident in the R18 and R19 regions of the side scatter versus yellow fluorescence dot plot. The population within the R18 region denoted dissolved NR, while the population within the R19 region indicated NR aggregation. After the filtration process, a significant reduction in the population of NR aggregation. The removal efficiencies for aggregated NR were 58.0%, 83.8%, and 96.8% for the 0.22 μm GF filter, 0.22 μm MCE filter, and 0.22 μm nylon filter, respectively. Correspondingly, the background noise within the R15 region, where NR-stained microplastics are situated, was determined to be 1.60×10^3^, 1.24×10^4^, and 1.11×10^5^ particles/mL, respectively. Our findings suggest that the majority of NR aggregation can be effectively eliminated through filtration with the 0.22 μm filter, with the 0.22 μm nylon filter demonstrating the most favorable performance in reducing NR aggregation. However, it is noteworthy that it also introduces a higher quantity of particulate impurities that might interfere with microplastic counting accuracy. Consequently, we employed the 0.22 μm GF filter to eliminate NR aggregation from the NR stock solution.

### 3.2 Optimal counting region

**FIGURE 2:**
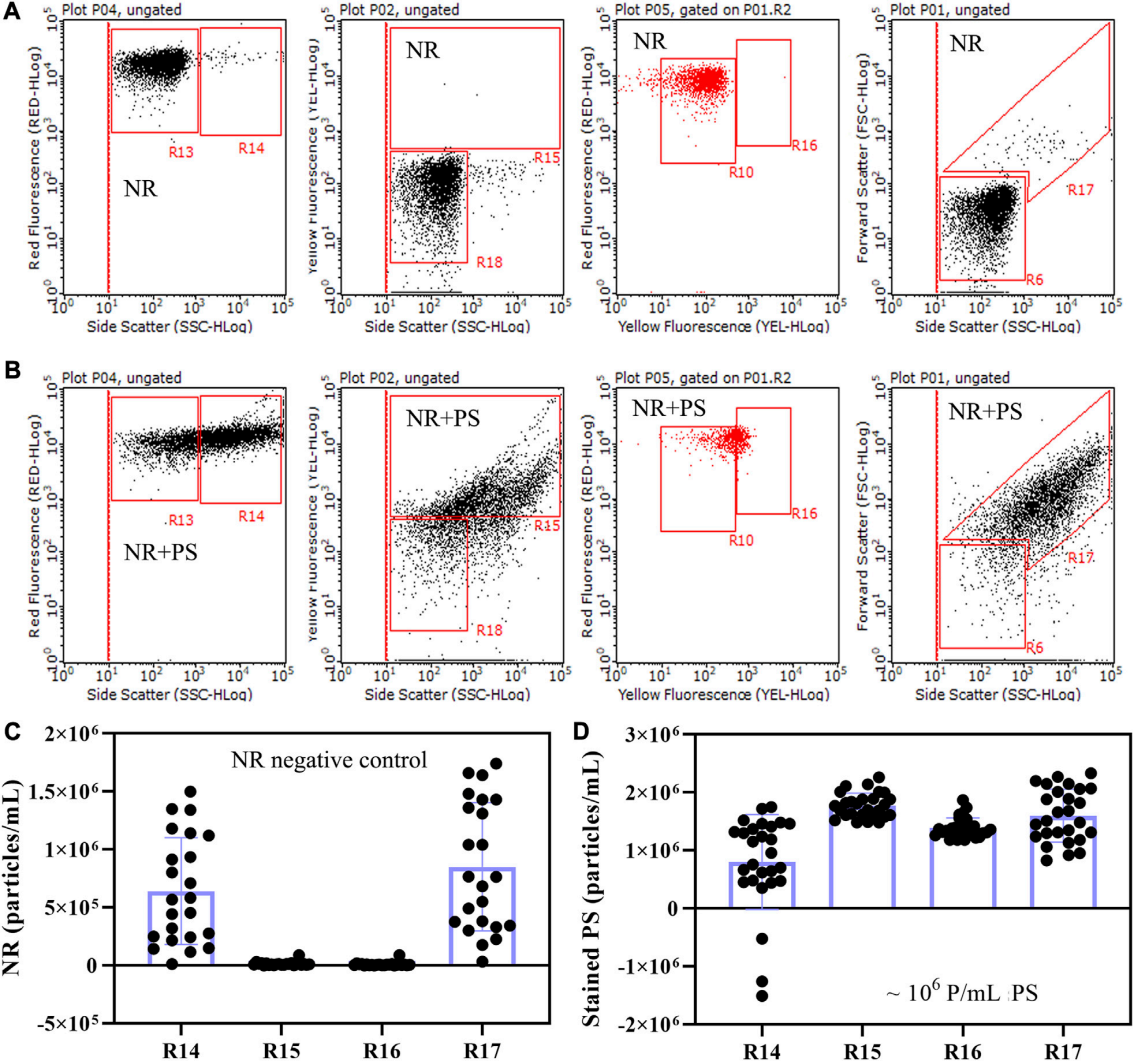
Comparison of four distinct dot plots involving NR **(A)** and NR-stained mixed PS microspheres with particle sizes ranging from 400 nm to 40 μm **(B)**. These dot plots and their corresponding counting region include side scatter versus red fluorescence (R14), side scatter versus yellow fluorescence (R15), yellow fluorescence versus red fluorescence (R16), and side scatter versus forward scatter (R17). The NR staining was in a 30% DMSO solution at room temperature. The populations residing within the R14, R15, R16, and R17 regions represent stained microplastics. The particle concentrations within these regions were assessed in 23 replicates of NR negative controls through flow cytometry **(C)**. Additionally, the particle concentration of 26 replicates of mixed PS microspheres **(D)** was derived by subtracting the particle concentration of NR-stained mixed PS microspheres **(B)** from the particle concentration of NR negative control **(A)** within the R14, R15, R16, and R17 regions.

In this investigation, the optimal counting region was determined through a comparative analysis of NR-stained microplastic counting results across four distinct dot plots. These dot plots encompassed the side scatter versus red fluorescence, side scatter versus yellow fluorescence, yellow fluorescence versus red fluorescence, and side scatter versus forward scatter. The delineation of counting regions specific to NR-stained microplastics was realized in the form of R14, R15, R16, and R17 regions (Figures 2A, B). The counts of particles within the R14, R15, R16, and R17 regions were tallied across 23 replicates of NR negative controls, resulting in average particle concentrations of 6.42×10^5^, 1.33×10^4^, 9.24×10^3^, and 8.50×10^5^ particles/mL, respectively (Figure 2C). These results highlighted that the background noise stemming from NR within the R15 and R16 regions was notably lower compared to that within the R14 and R17 regions. Furthermore, an analysis of 26 replicates involving PS microsphere liquid samples was conducted. This analysis involved subtracting NR background noise from counting results of stained PS microspheres within the R14, R15, R16, and R17 regions. The calculated particle concentration ranges for PS microspheres were as follows: R14 (−1.51×10^6^ to 1.75×10^6^ particles/mL), R15 (1.48×10^6^ to 1.79×10^6^ particles/mL), R16 (1.18×10^6^ to 1.87×10^6^ particles/mL), and R17 (8.27×10^5^ to 2.33×10^6^ particles/mL) (Figure 2D). Notably, the discrepancy in particle concentration between replicates of stained PS microspheres, calculated by the R15 and R16 regions, was smaller than that observed when using the R14 and R17 regions. This observation underscores the accuracy of the former and casts doubt on the reliability of the latter. Our findings underline that the optimal microplastic particle counting should be predicated on the R15 and R16 regions, as opposed to the R14 and R17 regions.

The substantial variance observed between replicates utilizing the R14 and R17 counting regions can likely be attributed to the interference stemming from background noise generated by agglomerated NR. To elucidate the contrasting signals emitted by aggregated NR and stained microplastics, we conducted a comparative analysis employing four distinct dot plots for both aggregated NR and NR-stained PS (Supplementary Figure S5). Through an analysis of variations in red fluorescence, yellow fluorescence, forward scatter, and side scatter among aggregated NR and NR-stained PS, we have identified the difference between agglomerated NR and stained microplastics. As NR transitions from a dissolved to an aggregated state, there is a simultaneous increase in forward scatter, side scatter, and red fluorescence values, while the yellow fluorescence remains relatively stable. In the case of NR-labeled PS, notable increments are observed across four parameters—forward scatter, side scatter, red fluorescence, and yellow fluorescence. The primary distinguishing factor between aggregated NR and stained microplastics centers around yellow fluorescence. The yellow fluorescence value of aggregated NR experiences marginal alteration, while that of NR-labeled PS undergoes a substantial increase. This phenomenon underscores the feasibility of discerning between the populations of aggregated NR and NR-stained microplastics through dot plots incorporating yellow fluorescence.

### 3.3 Optimizing NR staining

**FIGURE 3:**
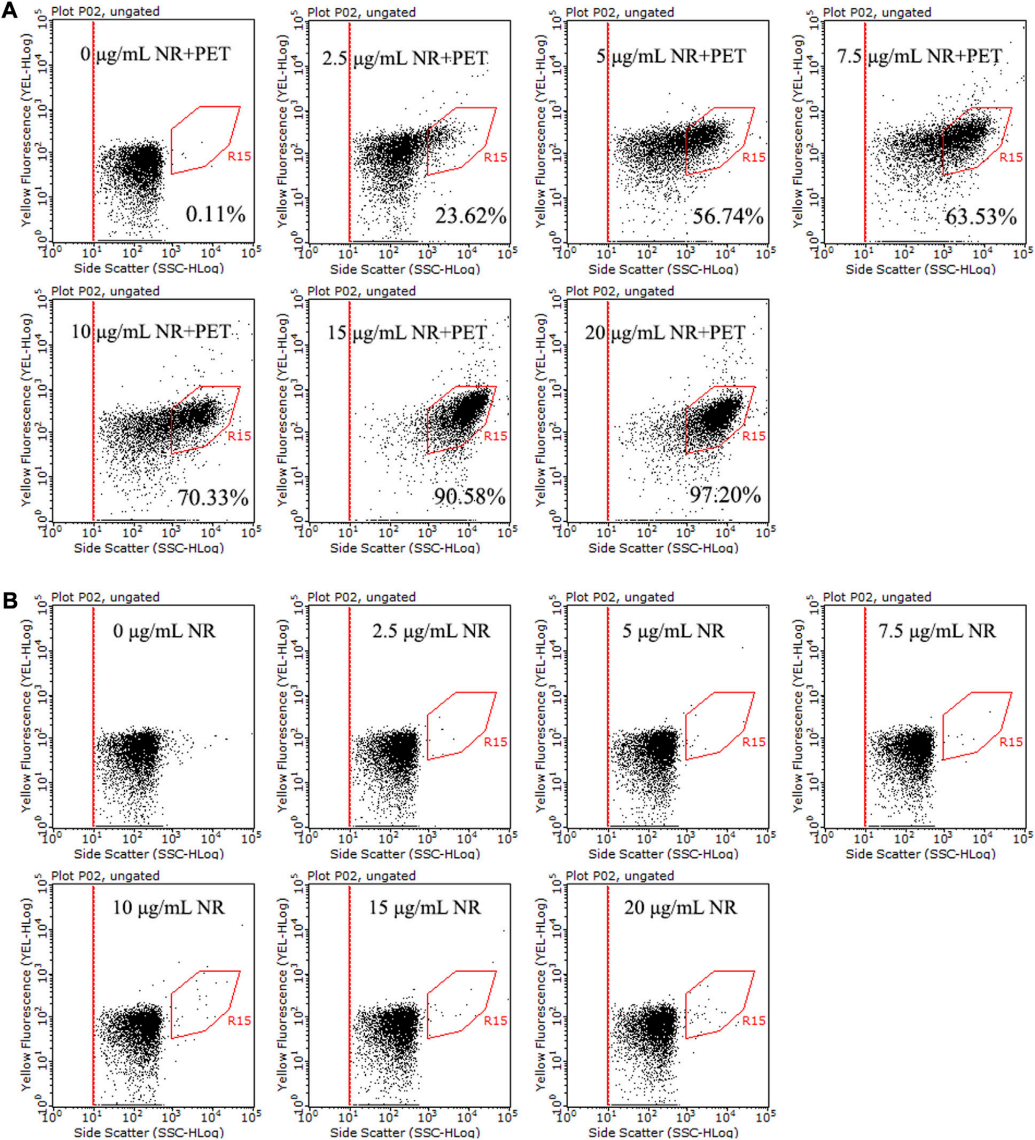
Dot plots of side scatter versus yellow fluorescence for NR stained PET (30 μm, ∼10^6^ particles/mL) **(A)** and NR negative control **(B)**, with different concentrations of NR (0–20 μg/mL) in 15% DMSO at room temperature for 10 min. The population in the R15 region represents stained microplastics.

This study explored the staining effect of gradient concentrations of NR on three types of microplastics, PET, PS, and PP. The proportion of stained PET was promoted with the increase of NR concentration (Figure 3), and the results of PE and PP showed the same trend (Supplementary Figures S6A, S6B). When the concentration of NR was up to 15 μg/mL and 20 μg/mL, more than 90% PET, PE, and PP were labeled. The above results show that the optimal concentration of NR is 15–20 μg/mL.

This study investigated the duration of NR-stained microplastics at the temperatures of 25°C and 60°C. The flow cytometry was used to analyze the staining effect of NR on PS microspheres for 10 min, 30 min, 1 h, and 2 h (Supplementary Figure S7). PS microspheres were labeled with NR in brown glass vials for 10 min at the temperatures of 25°C and 60°C. Stained PS still exhibited a strong fluorescence signal when NR was added for 2 h. Our results showed that NR has a good staining effect on PS under both room temperature and 60°C heating, which is a good dye for labeling microplastics. Additionally, we compared the staining of PS microspheres in plastic centrifuge tubes or glass vials at room temperature. In plastic centrifuge tubes, stained PS microspheres exhibited strong fluorescence signals within 60 min, while NR negative controls maintained strong fluorescence within 40 min, but lost fluorescence at 60 min (Supplementary Figure S8). Despite using the same staining solution (30% DMSO), the dissolution state of NR in plastic centrifuge tubes surpassed that in glass vials. The latter exhibited more pronounced agglomerated NR noise. Fortunately, within the side scatter versus yellow fluorescence dot plot, the noise signal from agglomerated NR and stained PS were distributed in separate regions, thereby causing minimal disruption to the counting of stained microplastics. For plastic samples amenable to analysis within a 40-min staining window, the choice between plastic-centric tubes or glass vials is viable. However, in scenarios involving microplastic samples requiring staining durations exceeding 40 min, the preference leans toward using glass vials for the NR staining.

### 3.4 The detection limit for plastic particles

**FIGURE 4:**
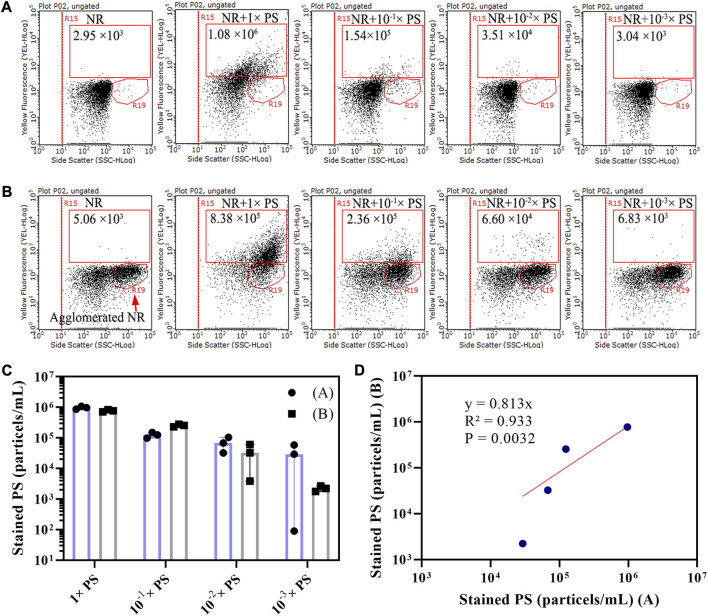
Dot plots of side scatter versus yellow fluorescence for 10-fold gradient-diluted mixed PS microsphere (400 nm–40 μm) liquid samples that had been NR stained. Two scenarios were considered: PS stained with 15 μg/mL of dissolved NR **(A)**, and PS stained with 15 μg/mL NR that contained agglomerated NR **(B)**. The NR staining was conducted in a 20% DMSO solution at room temperature. The population within the R15 region is indicative of stained PS, and the population within the R19 region represents agglomerated NR. To calculate the concentration of stained PS, we utilized the counting results derived from the R15 region of both scenarios A and B **(C)**. This calculation involved subtracting NR background noise from the counting results of stained PS microspheres within the R15 region. A linear relationship was established between the concentrations of stained PS microspheres derived from scenarios A and B **(D)**.

A mixed PS microsphere (400 nm–40 μm) liquid sample was diluted in 10 times gradient to explore the optimal detection concentration of microplastics by flow cytometry. In this study, we examined two scenarios: PS microspheres stained with dissolved NR or microspheres containing partially agglomerated NR. Under the first scenario, the particle concentration of the population in the R15 region of stained mixed PS microspheres (400 nm–40 μm) liquid sample and their 10 times gradient diluted samples were 1.08×10^6^, 1.54×10^5^, 3.51×10^4^, and 3.04×10^3^ particles/mL, respectively (Figure 4A). In the second scenario, the corresponding particle concentrations were 8.38×10^5^, 2.36×10^5^, 6.60×10^4^, and 6.83×10^3^ particles/mL, respectively (Figure 4B). The particle concentration of NR-stained PS was calculated by subtracting the particle concentration of the R15 region of the NR negative control. The particle concentrations of the two groups of measurement results were closed, with a good linear correlation (Figures 4C, D). Since the particle concentration of NR background noise is 10^3^–10^4^ particles/mL (Figure 2C), it could interfere with the identification of stained microplastics when the particle concentration of microplastics was less than 10^4^ particles/mL. Our results show that the optimal detection concentration of plastic particles is 10^5^–10^6^ particles/mL using flow cytometry.

### 3.5 Staining effect of NR on microspheres with different particle sizes

To explore the staining effect of NR on plastic particles with different sizes, stained PS microspheres with different particle sizes were analyzed under a scanning laser confocal microscope. The results demonstrated that both microplastics and nanoplastics were amenable to NR labeling (Supplementary Figure S9). Furthermore, a notable trend emerged: the fluorescence intensity increased with the size of the plastic particles. The results indicate that NR could serve as a fluorescent staining dye for plastic particles of various sizes.

**FIGURE 5:**
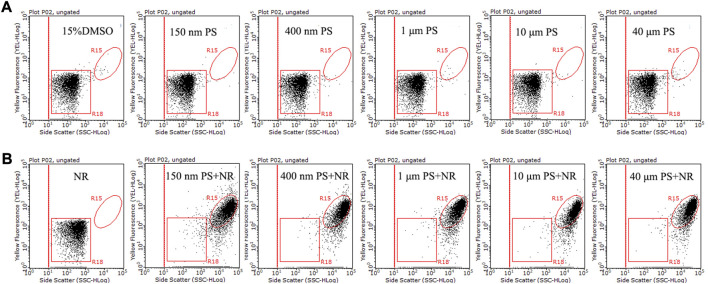
The side scatter versus yellow fluorescence dot plots of 15% DMSO and five different particle size PS microspheres (150 nm, 400 nm, 1 μm, 10 μm, and 40 μm) without **(A)** and with **(B)** 15 μg/mL NR. Following a 10-min NR staining in a 15% DMSO solution at room temperature, PS microsphere liquid samples were subjected to analysis using a flow cytometer. The particle concentration of five different particle sizes of PS microspheres was ∼10^6^ particles/mL. The R18 region represents dissolved NR, unstained PS microspheres, and background noise, while the population in the R15 region is indicative of NR-stained PS microspheres.

Five particle sizes (150 nm, 400 nm, 1 μm, 10 μm, and 40 μm) of PS microspheres were used as standard samples for flow cytometry analysis. The populations of PS microspheres (150 nm–40 μm) without NR and the 15% DMSO solution were distributed in the R18 region, which was in the bottom left of the dot plots (side scatter versus yellow fluorescence) (Figure 5A). The population of NR without PS was also distributed in the same region (Figure 5B). The populations of stained PS microspheres (150 nm–40 μm) were located in the R15 region, which was clearly separated from the population of unstained PS microspheres and NR (Figure 5B). Previous studies have shown that the detection range of flow cytometry for plastic particles is 200 nm–50 μm (Kaile et al., 2020). However, our result showed that 150 nm PS microspheres with NR also could be detected by flow cytometry, it may be because the particle size of 150 nm PS microspheres can be increased by NR staining. Our results here indicated that flow cytometry could analyze nanoplastics and microplastics with a range of 150 nm–40 μm.

### 3.6 Staining effect of NR on different types of plastic particles

**FIGURE 6:**
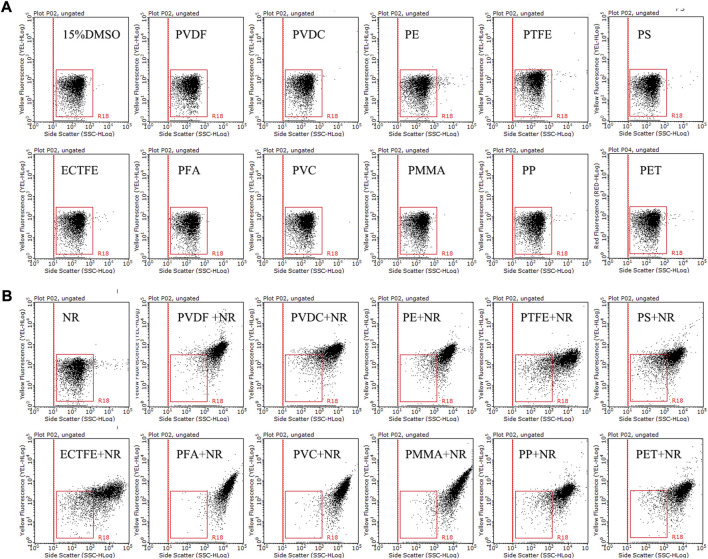
The side scatter versus yellow fluorescence dot plots of 15% DMSO and 11 different types of microplastics without **(A)** or with **(B)** NR staining. Tested microplastics, including PVDF (12 μm), PVDC (12.5 μm), PE (20 μm), PTFE (3 μm), PS (10 μm), ECTFE (23 μm), PMMA (≤50 μm), PVC (≤50 μm), PFA (23 μm), PP (20 μm), and PET (30 μm), were incubated with 15 μg/mL NR in 15% DMSO at room temperature for 10 min. The R18 region represents dissolved NR and background noise, while the population outside the R18 region and located in the upper right corner of the dot plot is stained microplastics.

To identify the staining effect of NR on different types of plastic particles, flow cytometry analysis was applied to detect 11 types of microplastics, including PVDF, PVDC, PE, PTFE, PS, ECTFE, PMMA, PVC, PFA, PP, and PET. The populations of 11 types of microplastics without NR staining were in the R18 region, distributed in the bottom left part of the dot plots (side scatter versus yellow fluorescence) (Figure 6A), while the counterparts with NR staining were located in the top right corner of the dot plots (Figure 6B). Our findings indicated a distinct separation between the population of NR-stained microplastics and the populations of both unstained microplastics and NR in the dot plots. This observation strongly suggests the successful staining of all 11 types of microplastics with NR. Our findings validate the viability of employing NR as a dye to label the majority of plastic particles for flow cytometry analysis.

### 3.7 Separation of microplastics and nanoplastics

**FIGURE 7:**
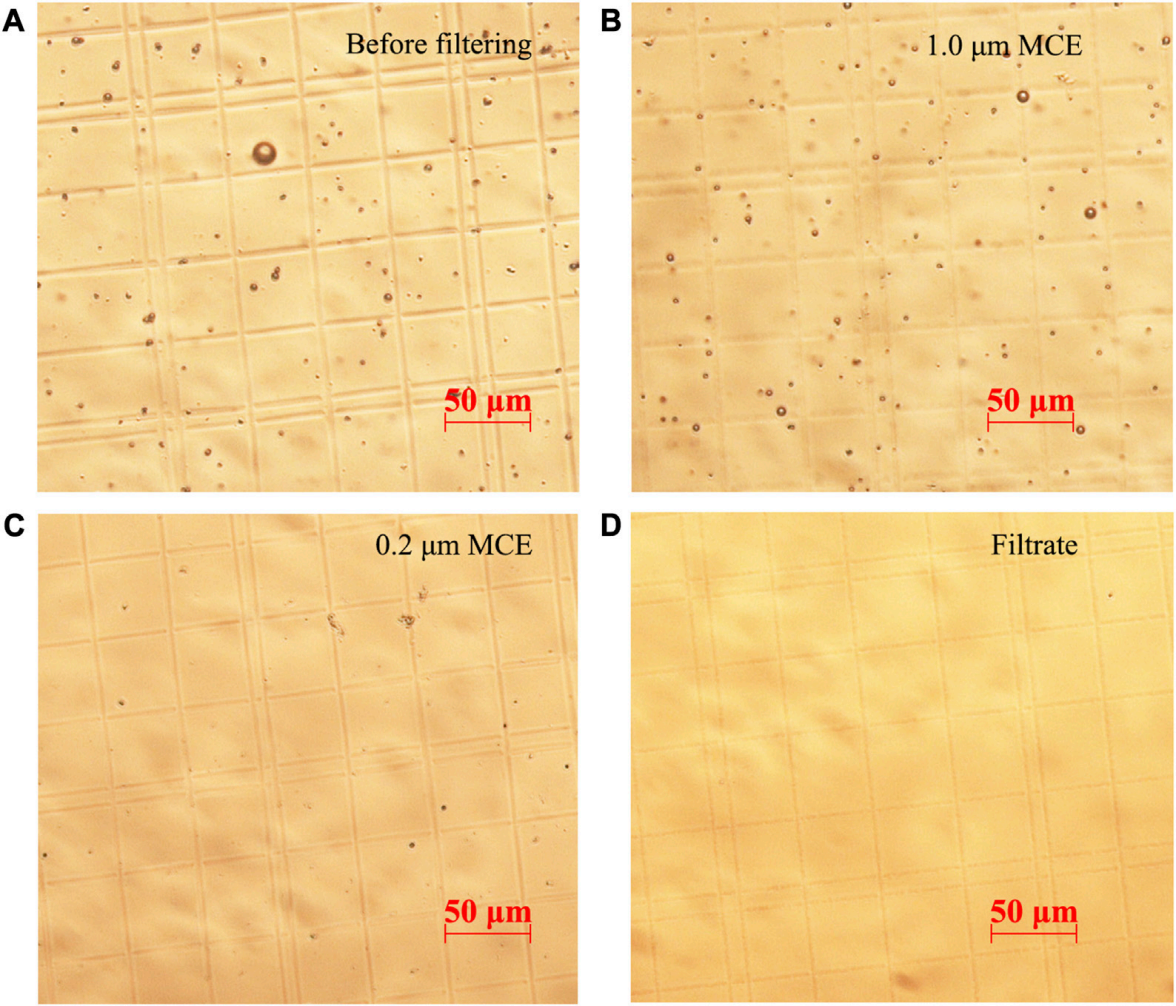
Microscopic images of the PS microspheres liquid samples screened by multistage filtration, including mixed PS microspheres (400 nm–40 μm) before filtration treatment **(A)**, PS microspheres intercepted by 1.0 μm filter membranes **(B)**, PS microspheres intercepted by 0.2 μm filter membranes **(C)**, and the filtrate of 0.2 μm filter membranes **(D)**.

400 nm, 10 μm, 1 μm, and 40 μm PS microspheres were mixed in 15% DMSO to prepare mix plastic particle liquid samples, which were sieved through 50 μm stainless steel meshes, 1.0 μm MCE filter membranes, and 0.2 μm MCE filter membranes orderly. Unfiltered samples contained PS microspheres with different particle sizes (Figure 7A). The particle size of PS microspheres intercepted by 1.0 μm filter membranes was 1.0–50.0 μm (Figure 7B). The particle size of PS microspheres intercepted by 0.2 μm filter membranes was about 0.2–1 μm (Figure 7C). The particle size of PS microspheres in the filtrate was less than 0.2 μm and could hardly be observed using a microscope (Figure 7D). The results showed that the microplastics with various particle sizes could be separated by multistage filtration.

We compared the recovery efficiency of three different kinds of filter membranes for plastic particles, including MCE filter membranes, PC filter membranes, and GF filter membranes. The recovery efficiency of MCE membranes, PC membranes, and GF membranes for plastic particles was 82.58%, 51.64%, and 23.84% respectively (Supplementary Figure S10), indicating that the MCE membranes had the highest recovery efficiency for plastic particles among these three kinds of filter membranes. The impurities in the plastic particles intercepted by the MCE filter membranes and PC filter membranes were less, but a lot of glass fiber fragments were brought in the plastic particles intercepted by GF filter membranes (Supplementary Figure S11). The above results show that MCE filter membranes are fit for filtering concentrated plastic particles.

The recovery efficiency for plastic particles of three different elution methods was compared in this study. The recovery efficiency for plastic particles of vortex, ultrasonication, and homogenization were 92.06%, 90.31%, and 90.72%, respectively, indicating that the recovery efficiency of the three elution methods for plastic particles was similar, all with more than 90% of the recovery efficiency (Supplementary Figure S12). The impurities in the plastic particles eluted by vortex and ultrasonication were less, while the impurities in the plastic particles eluted by homogenization were more (Supplementary Figure S13).

We used flow cytometry to analyze the damage effects of the three elution methods on the MCE filter membranes. Our results showed that homogenization caused the most damage to the filter membrane, followed by vortex and ultrasonication the least (Supplementary Figure S14). The broken tiny particles of the MCE filter membranes could be labeled by NR and would cause interference with the plastic particle counting. Therefore, we chose ultrasonication as the elution method. To remove the interference caused by MCE filter membrane debris, the suspension of the filter membranes filtered with ultrapure water was performed as the blank control of plastic particle liquid samples collected by the filter membrane.

### 3.8 Detection of simulated water samples

**FIGURE 8:**
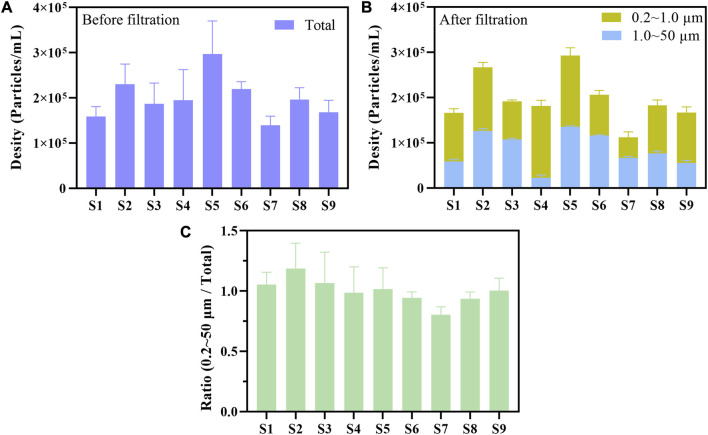
Flow cytometry was used to measure the particle concentration of microplastics and nanoplastics in 3 ultrapure water samples (S1–S3), 3 tap water samples (S4–S6), and 3 lake water samples (S7–S9) before **(A)** or after multistage filtration **(B)**. The plastic particles were separated into microplastics (1.0–50.0 μm) and nanoplastics (0.2–1.0 μm) by multistage filtration **(B)**. The particle concentration of microplastics and nanoplastics was counted by the R15 region of side scatter versus yellow fluorescence dot plot. The ratio of the sum of microplastics and nanoplastics (0.2–50 μm) in the total plastic particles was calculated based on the counting results **(C)**.

The flow cytometry was used to count the total plastic particles, the plastic particles (1.0–50.0 μm) intercepted by 1.0 μm filter membranes, and the plastic particles (0.2–1.0 μm) intercepted by 0.2 μm filter membranes of 9 simulated water samples. The particle concentration of total plastic particles before multistage filtration for 9 water samples was 1.39 × 10^5^–2.97×10^5^ particles/mL (Figure 8A). The particle concentration of microplastics (1.0–50.0 μm) was 2.27 × 10^4^–1.16×10^5^ particles/mL, and the counterpart of nanoplastics (0.2–1.0 μm) was 4.53 × 10^4^–1.59×10^5^ particles/mL (Figure 8B). The ratio of the sum of microplastics (1.0–50.0 μm) and nanoplastics (0.2–1.0 μm) in the total plastic particles of 9 water samples was 0.80–1.19 (Figure 8C). Our results indicated that we could turn the particle counting of microplastics and nanoplastics respectively into reality by combining multistage filtration and flow cytometry.

We also compared the results of flow cytometry and microscope counting methods on 15 standard microplastic samples. The results are shown in Supplementary Table S2. The particle concentration of microplastic particles measured by flow cytometry is 0.81–45.66 times that measured by microscope counting. The particle size range that can be detected by flow cytometry is 0.2–50 μm (Kaile et al., 2020), while microplastics with a size of less than 1 μm hardly can be counted under the light microscope (Adhikari et al., 2022). This may help to explain why the results of flow cytometry are generally greater than those of microscope counting. The results imply that the flow cytometry could be more accurate and reliable than the optical microscope in counting the number of microplastic and nanoplastics.

## 4 Discussion

### 4.1 Removal of NR aggregate and precipitation

NR precipitation is one of the important factors that affect the accuracy of flow cytometry counting microplastics. NR is a lipophilic dye, which is very sensitive to the solution and has strong fluorescence only in hydrophobic solution (Greenspan et al., 1985). In the water, NR is prone to aggregate and its fluorescence intensity is very weak (Supplementary Figure S15). Although it has good selectivity for microplastics (Shim et al., 2016; Shruti et al., 2022), the fluorescence and solubility of NR are greatly affected by the fact that flow cytometry can only be performed in aqueous solutions. Precipitated NR can form solid particles that are easily recognized by flow cytometry as microplastics, resulting in false positives (Kaile et al., 2020). The results of this study confirm the truth that the aggregated NR population and the stained microplastics population are distributed in the same area of the side scatter versus red fluorescence dot plot (Figure 2; Supplementary Figure S5). This issue greatly interferes with the accuracy of flow cytometry in counting microplastic particles.

NR precipitation may come from two processes: 1) Due to the high concentration of NR in the stock solution, some NR is not dissolved in the solvent of DMSO; 2) NR agglomerates in aqueous solutions while adding NR to the solution. For the first process, we took the following measures to reduce agglomerated NR, including selecting appropriate solvents, ultrasonic treatment, and filtration with a 0.22 μm filter. Our results showed that the filtration could effectively reduce the precipitation of NR (Supplementary Figure S4), indicating that there is insoluble NR in the NR stock solution. So it is necessary to remove the precipitation of NR from the NR stock solution through filtration. For the second process, we improved the solubility of NR in the solution by optimizing the solution. 10% DMSO (Kaile et al., 2020) and 0.1% Tween 20 (Tse et al., 2022) were used as the solutions for the flow cytometry of microplastics. We compared the dissolution of NR in 10% DMSO and 0.1% Tween 20, and the results showed that NR dissolved better in 10% DMSO than in 0.1% Tween 20 (Supplementary Figure S3).

Due to the tendency of NR to form minor aggregates in 10% DMSO, there arises a potential interference with the precision of microplastic counting. To address this issue, we successfully mitigated the aggregates by enhancing the DMSO proportion to a range of 15%–30%. We found that different batches of NR stock solution have different dissolution states in 15% DMSO, and some batches of NR may form aggregates in 15% DMSO. However, the solubility of the same NR in 20%–30% DMSO was significantly improved. Therefore, we tested the dissolved state of NR in 15%, 20%, and 30% DMSO before we started microplastic staining, and then adjusted the proportion of DMSO in the solution for microplastic staining. In the case of the flow cytometer you are using can tolerate a higher proportion of DMSO solution, we strongly recommend using 20%–30% DMSO as the staining solution.

### 4.2 Selection of optimal counting region

Due to its hydrophobic nature, NR displays high instability within aqueous solution systems. Despite successfully mitigating the majority of agglomerated NR by increasing the DMSO to 20%–30% within the solution, a residual level of background noise from NR remains present within the side scatter versus red fluorescence dot plot region, where stained microplastics are concentrated. This issue has been previously highlighted due to signal overlap between NR and stained microplastics (Kaile et al., 2020). This persistent noise poses a minor yet significant challenge, particularly when quantifying microplastics of low concentrations.

Comparing the dot plots of different signal combinations of agglomerated NR and stained mixed PS microspheres, we observed distinctions between them. Transitioning from dissolved to aggregated states, NR showed significant increases in forward scatter, side scatter, and red fluorescence signals, while yellow fluorescence remains relatively stable (Supplementary Figure S5). Upon NR labeling of mixed PS microspheres, substantial enhancements are evident in forward scatter, side scatter, red fluorescence, and yellow fluorescence signals. These observations underscore the primary difference between the two entities, particularly in terms of yellow fluorescence intensity, which is notably greater in stained microplastics than in agglomerated NR. By utilizing this distinction in yellow fluorescence, we can effectively distinguish the populations of agglomerated NR and stained microplastics within dot plots incorporating yellow fluorescence. Such findings rationalize the higher accuracy of counting results derived from yellow fluorescence-associated dot plots compared to those without this component. In dot plots devoid of yellow fluorescence, the similar trends in red fluorescence, side scatter, and forward scatter between agglomerated NR and NR-stained microplastics result in their colocalization, inducing significant overlap. This scenario potentially leads to false positives or underestimation of microplastic particle concentrations. Our study underscores that, with proper counting region selection, even in the presence of agglomerated NR, staining microplastic counting remains relatively unaffected.

### 4.3 NR fluorescence maintenance time and staining container

Our results showed that the optimal concentration of NR is 15–20 μg/mL, which is slightly higher than the NR concentration (10 μg/mL) used in the previous study (Kaile et al., 2020; Tse et al., 2022). Currently, microplastics for flow cytometry analysis are stained at room temperature (Kaile et al., 2020; Tse et al., 2022). This study showed that microplastics incubated with NR in glass vials at a temperature of 25°C or 60°C can maintain fluorescence for at least 2 h, which is a good fit for flow cytometry analysis. Previous studies show that heating (50°C–75°C) can enhance the staining effect of NR on microplastic particles, enabling microplastic particles to maintain a stable fluorescence for a long period (over 2 months) (Lv et al., 2019; Konde et al., 2020). For some microplastics with low hydrophobicity, such as polyamide (PA) and polyester (PES) (Shim et al., 2016), that are difficult to label with NR at room temperature, it may be possible to try NR staining under heating conditions.

We also compared the staining effect of PS microspheres in plastic centrifuge tubes and glass tubes at room temperature, and the results showed that these two different materials containers would affect the solubility of NR in the 30% DMSO solution. We found that the fluorescence intensity sharply decreased when NR was placed in plastic centrifuge tubes without PS microspheres for more than 60 min, while NR still maintained strong fluorescence in glass tubes. We speculated that it might be due to the adsorption of NR by plastic centrifuge tubes, which affected the solubility of NR in 30% DMSO and was more conducive to its dissolution. With prolonged adsorption, the concentration of NR in 30% DMSO sharply decreases, gradually losing its fluorescence. Even though there was more aggregation of NR in glass vials compared to plastic centrifuge tubes, this minor aggregation of NR would have a minimal impact on microplastic counting when using the side scatter versus yellow fluorescence dot plot. NR can maintain long-term stable fluorescence intensity in glass vials, which further reinforces our recommendation for glass container usage during the microplastic staining process.

### 4.4 Difficulties and challenges

It is challenging to turn the application of flow cytometry in environmental microplastic and nanoplastic samples into reality. The observation we have made suggests a noticeable flaw within the context of flow cytometry analysis, where the stipulated detection limit stands at 10^4^ particles/mL (equivalent to 10^7^ particles/mL). Nonetheless, the concentration of microplastics in environmental water remains exceedingly low. The average abundance of microplastics in drinking water is 1–10 particles/L, and the counterpart in bottled water is 10^2^–10^4^ particles/L (Oßmann et al., 2018). The amount of microplastics in different freshwater ecosystems is lower, and the abundance of microplastics varies greatly, ranging from less than 0.1 particles/L to 10 particles/L (Thompson et al., 2009; Baldwin et al., 2016). The environmental water samples have to be concentrated 10^7^–10^8^ times to meet the detection requirements of flow cytometry. Filtering water samples with such a large concentration ratio will be time-consuming and laborious. This defect undoubtedly limits the application of flow cytometry in the detection of environmental microplastic samples. It is an important issue to be solved how to achieve the concentration of microplastics in environmental water efficiently.

It is another difficulty that realize the quantitative analysis of nanoplastic samples below 200 nm. The minimum size limit of ordinary flow cytometry is 200 nm (Kaile et al., 2020). Our results showed that 150 nm plastic particles can be analyzed by flow cytometry. This may be because NR wraps the outer surface of the plastic microspheres, increasing the particle size of the microspheres. However, microplastics below 100 nm cannot be analyzed by ordinary flow cytometry (Kaile et al., 2020). The particle size detection range of nanoflow cytometry for nanoparticles is 7–1,000 nm, covering the blind area of conventional flow cytometry. Nanoflow cytometry has been applied to characterize nanoparticles such as nanomaterials, subcellular structures, bacteria, viruses, and extracellular vesicles (Chen et al., 2019; Guo et al., 2021). The emergence of nanoflow cytometry makes it possible to count microplastic particles smaller than 100 nm. It is another significant challenge to enrich nanoplastics smaller than 100 nm from environmental water through conventional filtration because there is no suitable pore-size filter membrane.

Certain limitations are evident in the choices of filter membrane materials and elution methods within this study. To minimize eluent volume and enhance microplastic sample concentration, a recycling approach involving the shearing of MCE filter membranes and subsequent elution with 10%–30% DMSO solution was employed. However, this membrane shearing procedure may result in breakage and debris formation. Our investigation has demonstrated that NR labeling of MCE debris could introduce interference in microplastic counting (Supplementary Figure S14). Consequently, refining the elution technique or opting for alternative filter membrane materials becomes urgent to mitigate such interference. While one approach could involve direct elution of microplastics without filter membrane cutting, the efficiency of recovery upon elution remains a crucial consideration. Alternatively, selecting a filter membrane characterized by both high recovery efficiency and resistance to NR labeling could serve as a replacement for the MCE filter membrane. Our research results have shown that MCE has the highest recovery efficiency for microplastics, aligning with established literature findings (Tse et al., 2022). In situations when it is challenging to find a suitable filter membrane, a possible solution to reduce debris interference could be to explore better ways of elution.

## 5 Conclusion

We successfully improved the solubility of NR in the solution by enhancing the DMSO ratio. Also, we clarified the difference between the signals of aggregated NR and stained plastic particles lies in yellow fluorescence. By selecting the dot plot incorporating the yellow fluorescence and establishing a definitive counting region, we reduced the interference of aggregated NR, which is crucial for ensuring the accuracy of plastic particle counting through flow cytometry. Additionally, this study achieved the separation and concentration of microplastics (1.0–50.0 μm) and nanoplastics (0.2–1.0 μm) by multistage filtration. Combining multistage filtration, NR staining, and flow cytometry, a quantitative analysis method for microplastics and nanoplastics was established in this study. This study helps to improve the detection protocols of microplastics and nanoplastics by flow cytometry and could provide technical support for evaluating microplastics and nanoplastics pollution.

## Data Availability

The original contributions presented in the study are included in the article/Supplementary Material, further inquiries can be directed to the corresponding author.
