# Supplementary material for: Separation and flow cytometry analysis of microplastics and nanoplastics
Source: Front Chem. 2023 Sep 15;11:1201734. doi: 10.3389/fchem.2023.1201734 (PMC10540628; doi:10.3389/fchem.2023.1201734)
Supplement: Supplementary file 1 [file DataSheet1.pdf]

frontiers

Supplementary Material

Separation and flow cytometry analysis of microplastics and nanoplastics

Jingjing Li[1], Fuyi huang[1,2], Guohui Zhang[1,3], Zixing Zhang[1], Xian Zhang[1 *]

[1]Key Laboratory of Urban Environment and Health, Institute of Urban Environment, Chinese Academy of Sciences, Xiamen, 361021, China

[2]University of Chinese Academy of Sciences, Beijing, 100049, China

[3] College of Life Sciences, Fujian Agriculture and Forestry University, Fuzhou, 350002, China

*** Correspondence:** Xian Zhang: xzhang@iue.ac.cn

Table S1. Plastic particles used in this study

Plastic Type	Particle size	Company
PVDF	12 µm	SECCO, China
PVDC	12.5 µm	DuPont, USA
ECTFE	23 µm	DuPont, USA
PTFE	3 µm	DuPont, USA
PFA	23 µm	SECCO, China
PP	20 µm	SECCO, China
PE	20 µm	KPIC, Korea
PET	30 µm	DuPont, USA
PVC	≤ 50 µm	SECCO, China
PMMA	≤ 50 µm	SECCO, China
PS microspheres	40 µm, 10 µm, 1 µm	SECCO, China
PS microspheres	400 nm, 150 nm	Electrification, Japan

Table S2. Comparison of flow cytometry and microscope counting.

Sample No.	Flow cytometry (a, particles/mL)	Microscope (b, particles/mL）	Ratio (a/b)
**M1**	2.59×10^{5}	1.67×10^{5}	1.56
**M2**	2.58×10^{7}	1.74×10^{7}	1.48
**M3**	4.93×10^{7}	6.13×10^{7}	0.81
**M4**	2.11×10^{6}	2.53×10^{6}	0.83
**M5**	1.70×10^{7}	8.50×10^{6}	2.00
**M6**	2.26×10^{8}	1.04×10^{8}	2.17
**M7**	3.48×10^{7}	1.52×10^{7}	2.29
**M8**	4.95×10^{7}	1.55×10^{7}	3.19
**M9**	1.84×10^{8}	3.28×10^{7}	5.62
**M10**	1.47×10^{4}	1.29×10^{4}	1.14
**M11**	3.74×10^{4}	6.30×10^{3}	5.93
**M12**	1.69×10^{5}	3.70×10^{3}	45.66
**M13**	1.64×10^{5}	1.18×10^{4}	13.95
**M14**	1.27×10^{5}	1.17×10^{4}	10.93
**M15**	8.06×10^{4}	1.90×10^{3}	42.41

M1-M15: 15 standard microplastic liquid samples.

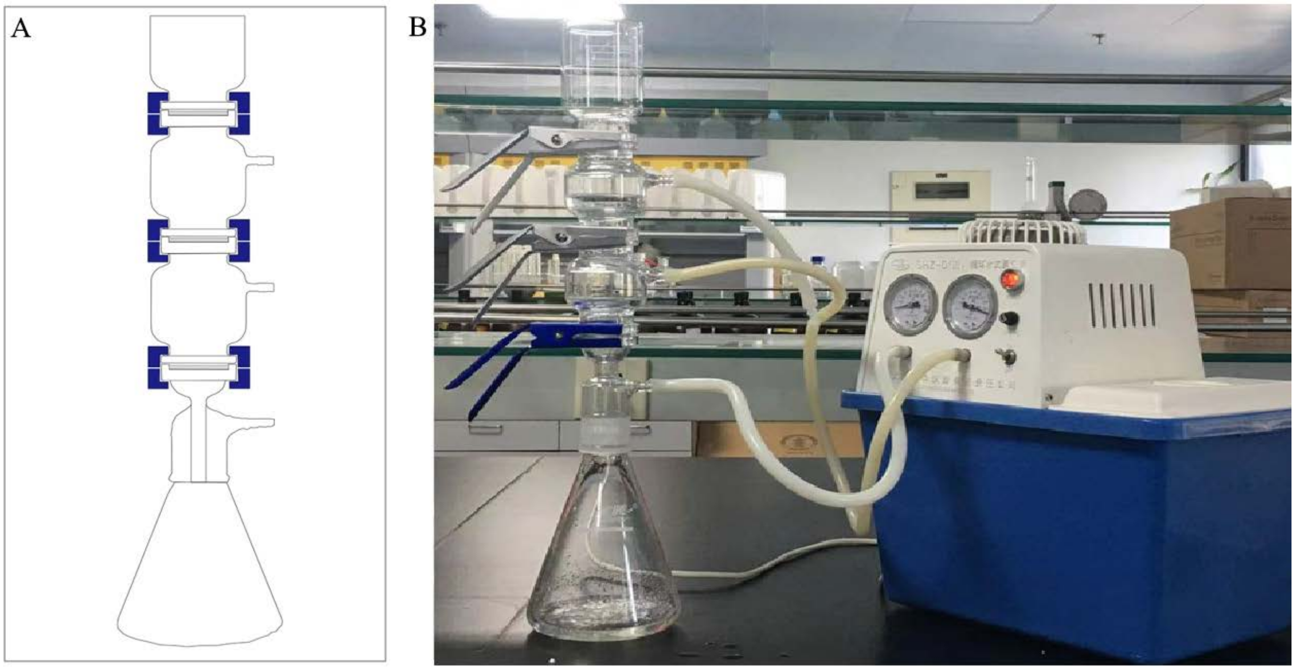

Figure S1 A grading filtration device was designed to separate microplastics and nanoplastics. The plastic liquid samples were sieved through 50 μm stainless steel mesh, 1.0 μm membrane filter, and 0.1 μm membrane filter, sequentially. 50 μm stainless steel meshes were used to remove substances with more than 50 μm size. 1.0 μm filter membranes were used to intercept 1.0–50 μm plastic particles. 0.2 μm filter membranes were used to intercept 0.2–1.0 μm plastic particles. The size of plastic particles in the filtrate of 0.2 μm filter membranes was 0–0.2 μm.

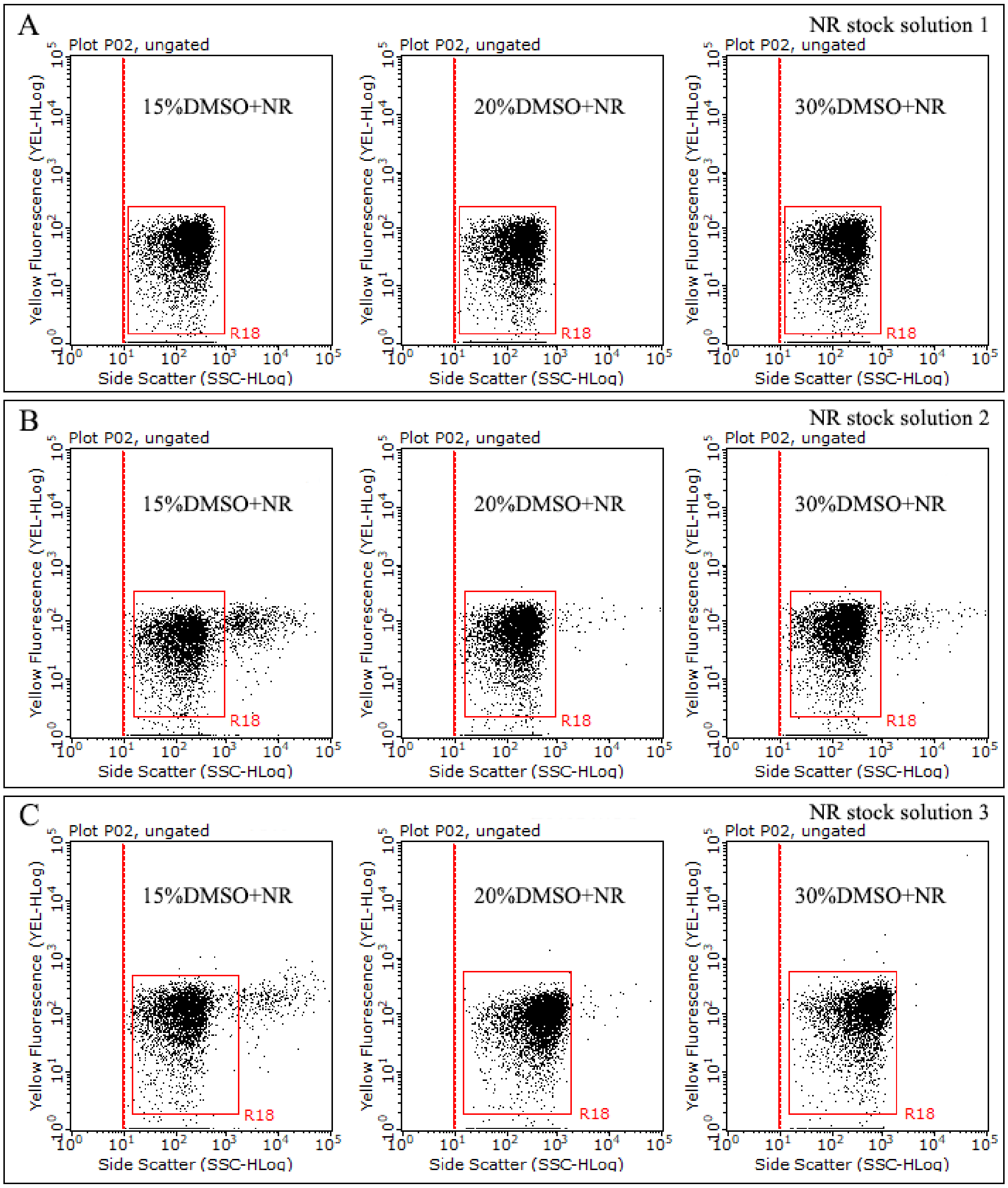

Figure S2 Comparison of the dissolution of three different NR stock solutions in 15%DMSO, 20%DMSO, and 30%DMSO. These three batches of NR stock solutions are marked as NR stock solution 1 (A), NR stock solution 2 (B), and NR stock solution 3 (C).

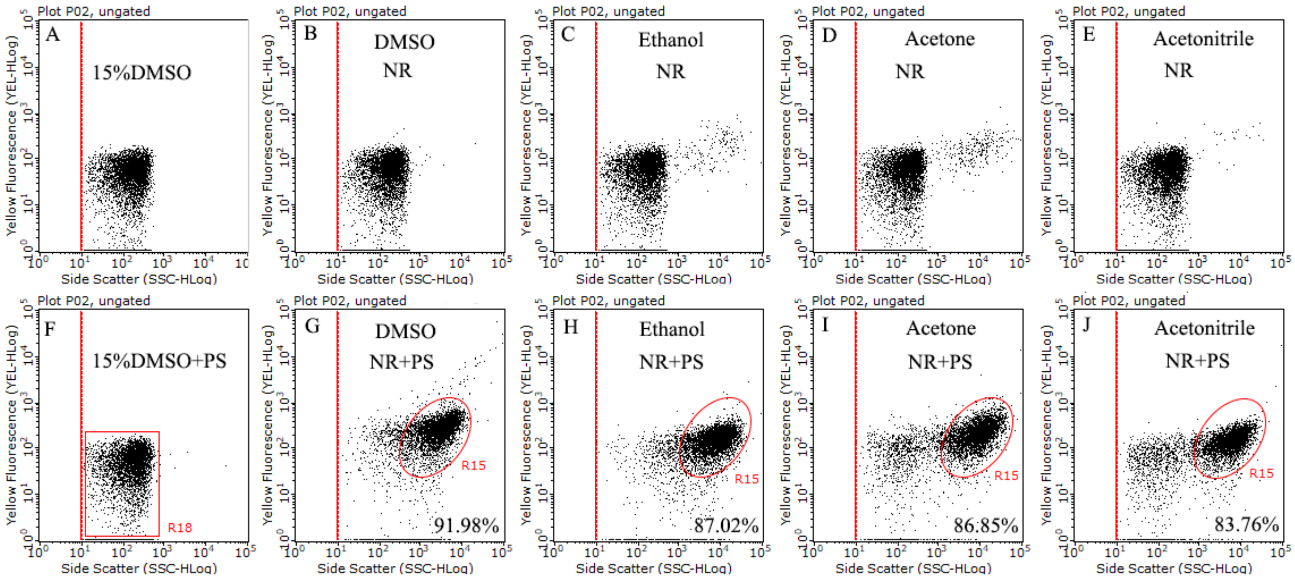

Figure S3 This study compared the population distribution of NR and stained PS (10 μm) in the dot plot of side scatter versus yellow fluorescence when preparing NR stock solution with four different solvents, including DMSO (B, G), ethanol (C, H), acetone (D, I), and acetonitrile (E, J). The NR staining was performed in the 15% DMSO solution (A). The staining efficiency is calculated by dividing the particle concentration of the R15 region by the particle concentration of the R18. The population in the R15 region represents stained PS, and the population in the R18 region represents unstained PS (F).

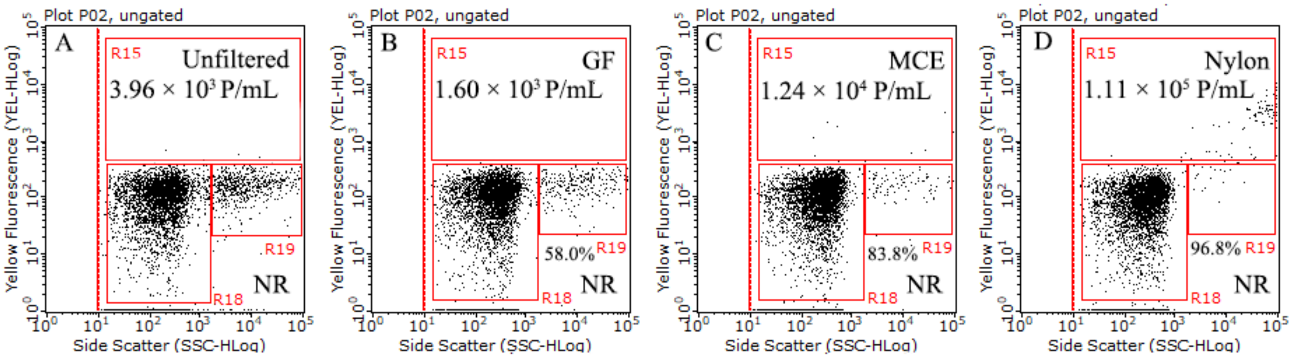

Figure S4 1 mg/mL NR stock solution (DMSO as solvent) was filtered through a 0.22 μm filter to remove the large particles of NR. 1.5 μL of 1 mg/mL NR stock solution was added into 98.5 μL of 30% DMSO, standing for 10 min at room temperature after vortex mixing, and immediately counted and analyzed using flow cytometry after vortex mixing again. We compared the dot plots of the NR population of unfiltered NR stock solution and NR stock solution filtered with 0.22 μm GF filter (B), 0.22 μm MCE filter (C), and 0.22 μm nylon filter (D). The population in the R15 region represents stained PS, the population in the R18 region represents dissolved NR, and the population in the R19 region represents the aggregation of NR. The removal efficiencies of the three different filters for agglomerated NR were 58.0%, 83.8%, and 96.8%. The background noise in the R15 region was 3.96×10^3 particles/mL, 1.60×10^3 particles/mL, 1.24×10^4 particles/mL, and 1.11×10^5 particles/mL, respectively.

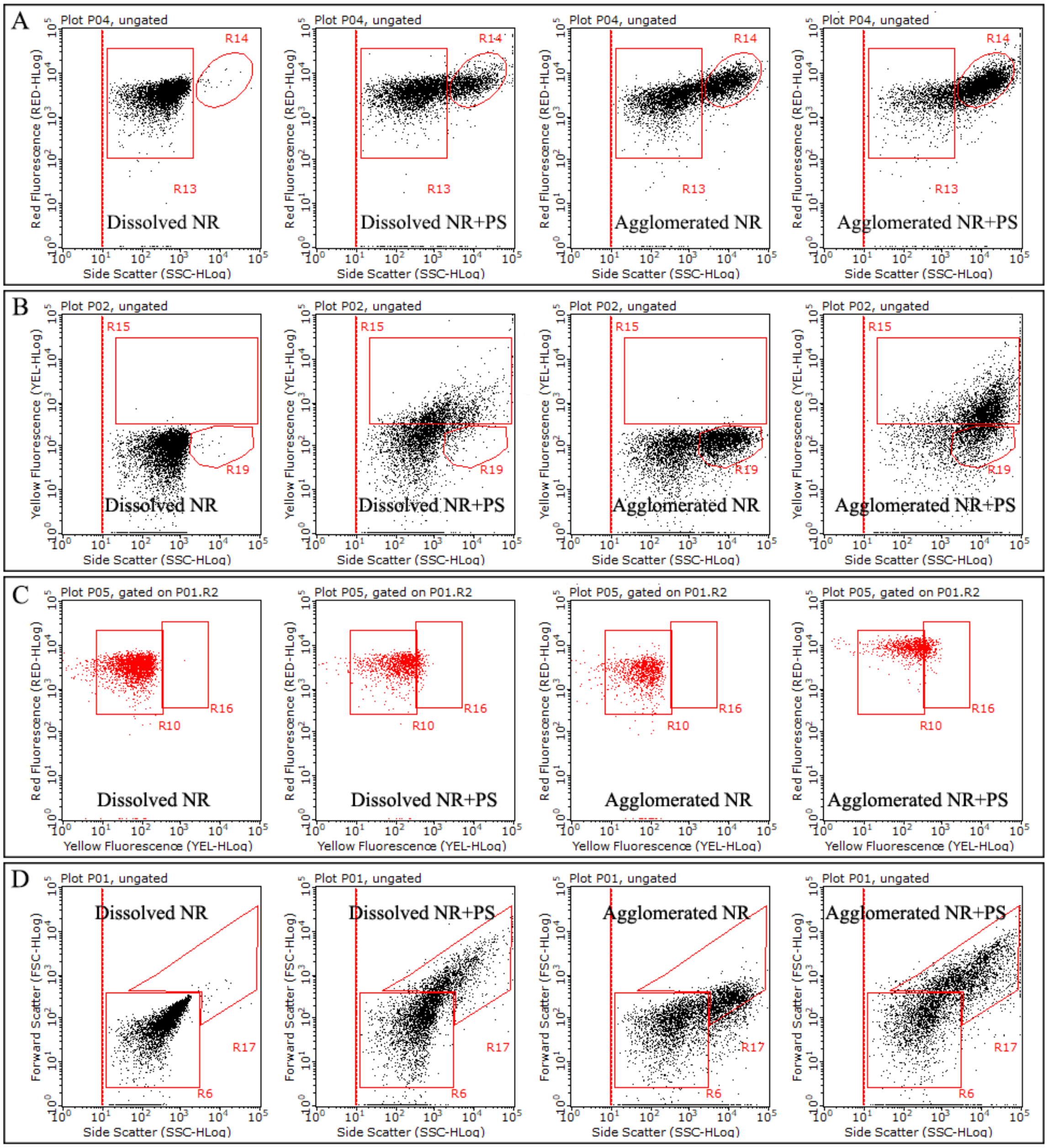

Figure S5 Comparison of the distribution of dissolved NR, agglomerated NR and stained mixing PS microspheres (150 nm–40 μm) in 4 different dot plots, including side scatter versus red fluorescence dot plot (A), side scatter versus yellow fluorescence dot plot (B), yellow fluorescence versus red fluorescence dot plot (C), and forward scatter versus side scatter dot plot (D). Mixing PS microspheres were stained with 15 μg/mL NR in 30% DMSO for 10 minutes. The populations in the R6, R10, and R13 regions represent dissolved NR and background noise, the population in the R14, R15, R16, and R17 regions represent stained microplastics, and the population in the R19 region represents agglomerated NR.

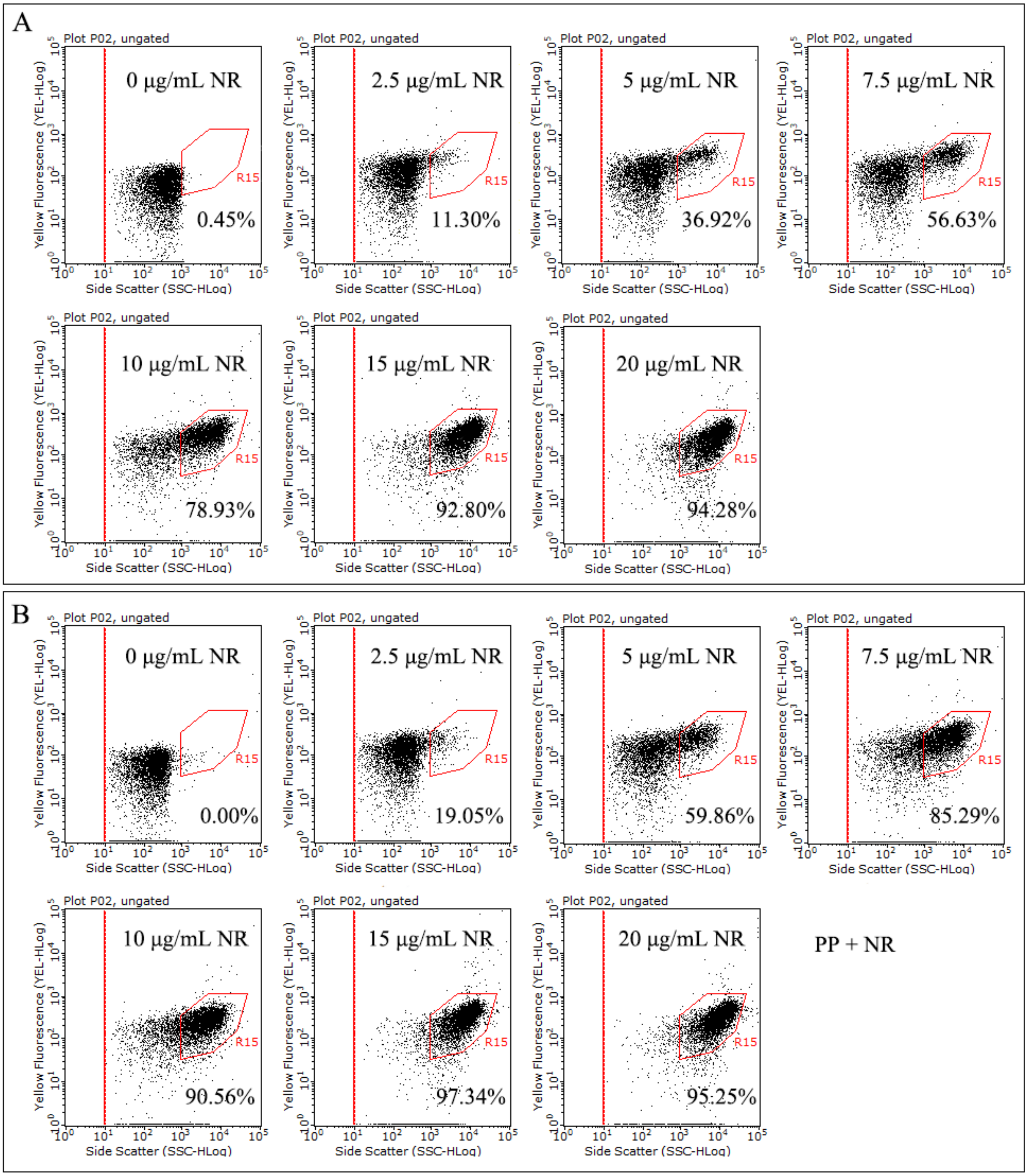

Figure S6 Staining effect of different concentrations of Nile Red on microplastic polyethylene (PE) (A) and polypropylene (PP) (B) was stained by different concentrations of NR (0–20 μg/mL) in 15% DMSO at room temperature for 10 min, which was analyzed by flow cytometry. 0–20 μg/mL NR in 15% DMSO was performed as negative control (C). The population in the R15 region represents stained microplastics.

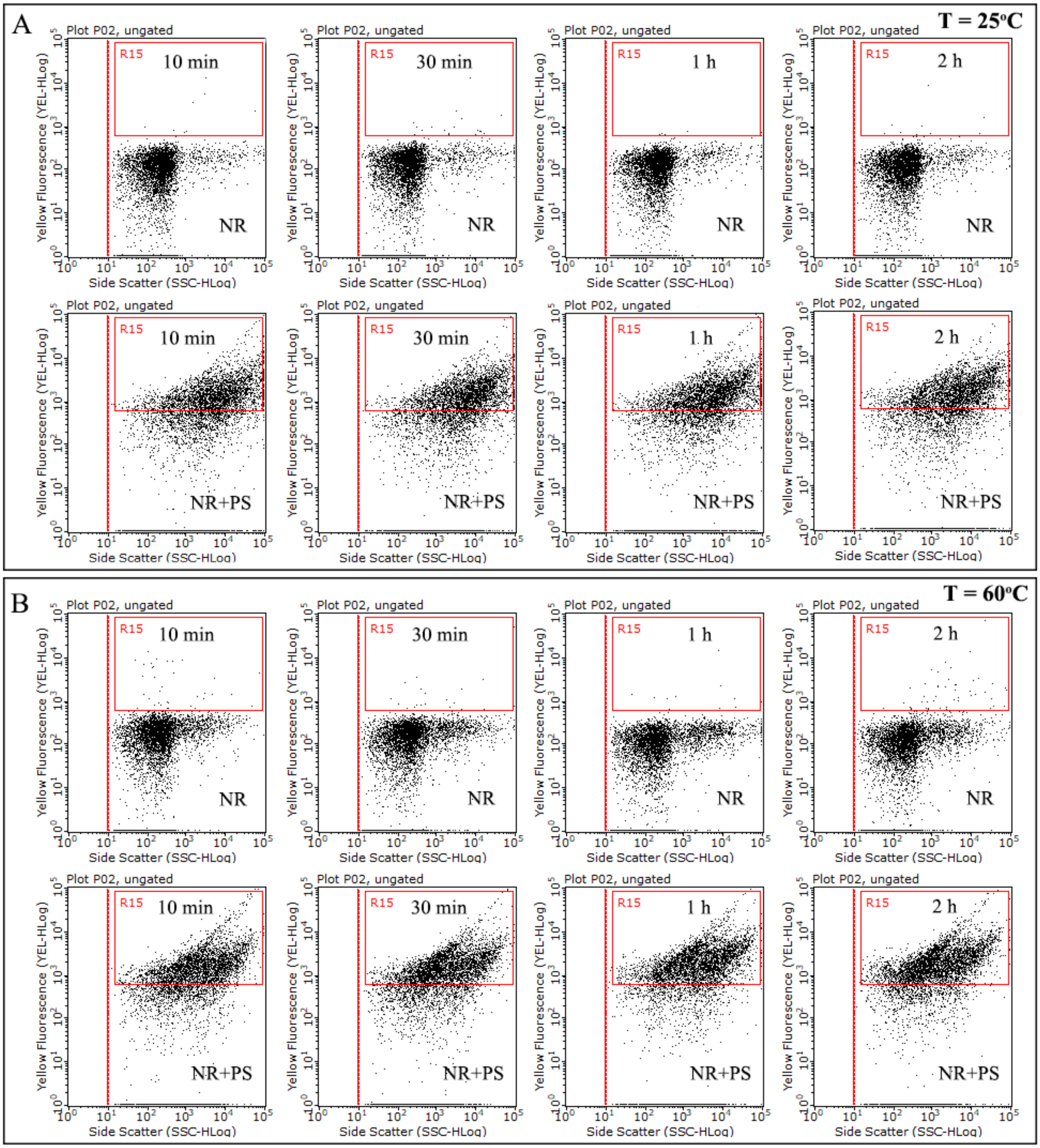

Figure S7 Mixing PS microspheres (150 nm–40 μm) were stained by NR with a final concentration of 15 μg/mL in 30% DMSO in 2 mL brown glass vials at a room temperature of 25℃ (A) or a temperature of 60℃ (B) for different staining times (10 min, 30 min, 1 h, and 2 h), which were analyzed by flow cytometry. The population in the R15 region represents stained microplastics.

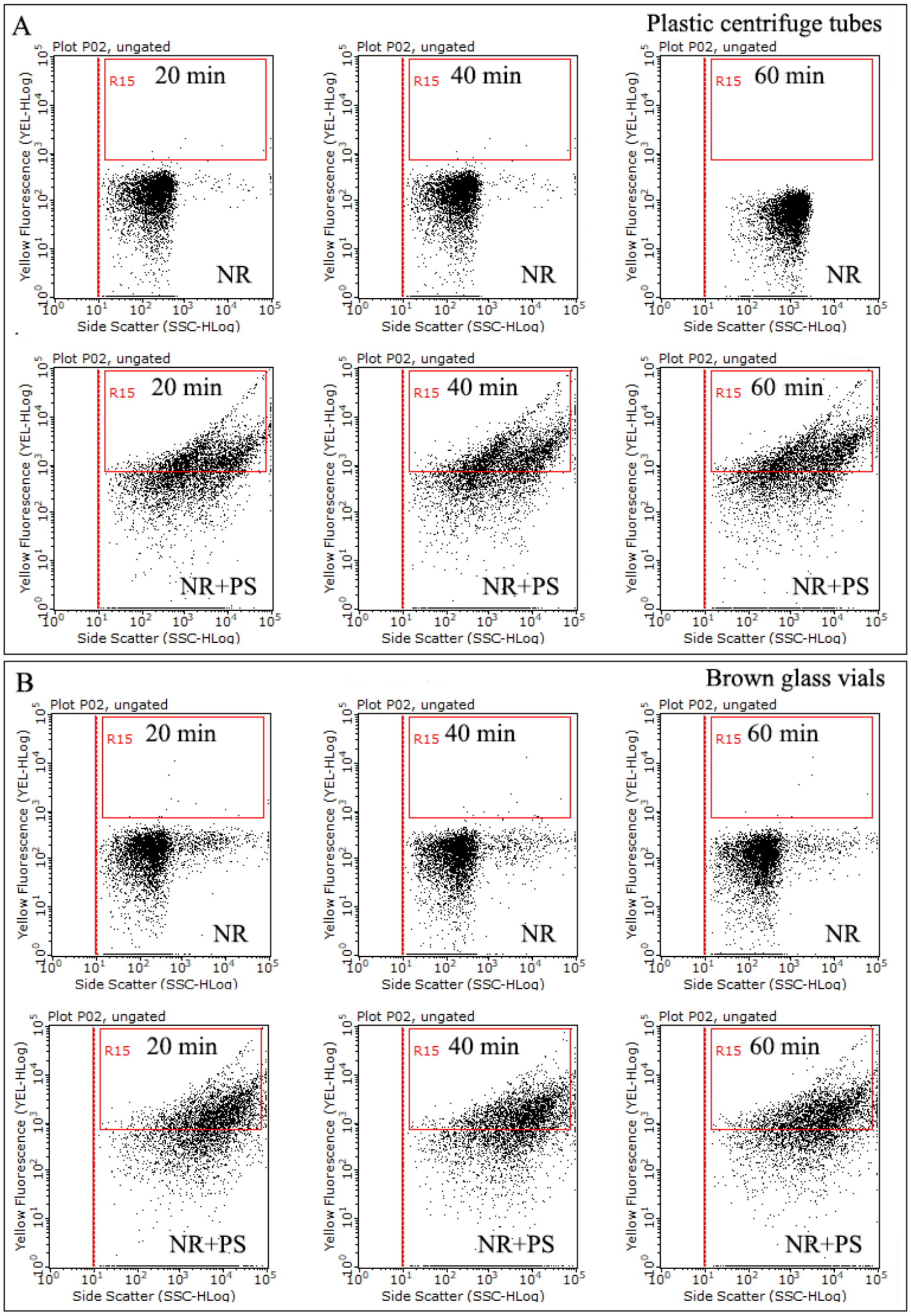

Figure S8 Mixing PS microspheres (150 nm–40 μm) were stained by NR with a final concentration of 15 μg/mL in a 30% DMSO solution in 1.5 mL plastic centrifuge tubes (A) or in 2 mL glass vials (B) at a room temperature for different staining times (20 min, 40 min, and 60 min), which were analyzed by flow cytometry. The population in the R15 region represents stained microplastics.

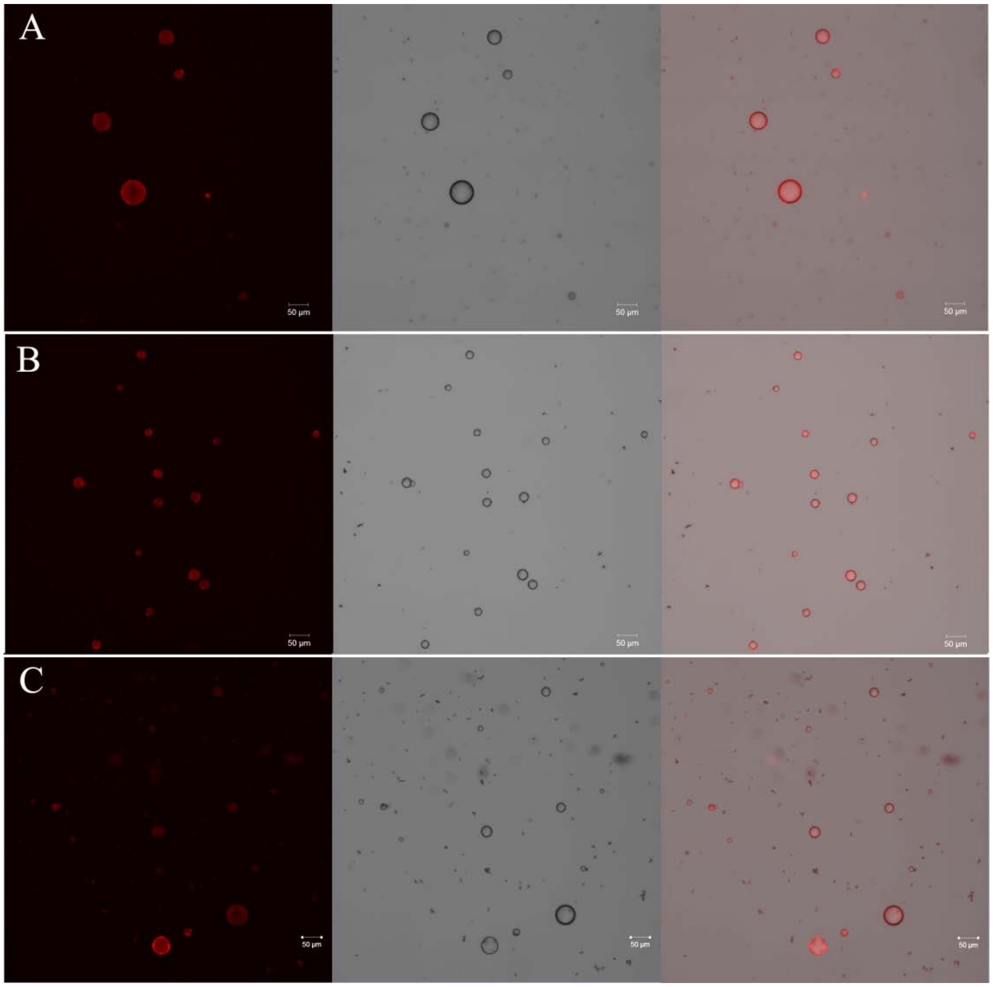

Figure S9 Scanning laser confocal microscopy imaging of stained PS microspheres. 20–40 μm PS microspheres (A), 10 μm PS microspheres (B), and 150 nm–40 μm mixed PS microspheres (C) were stained with 15 μg/mL NR at room temperature for 20 min.

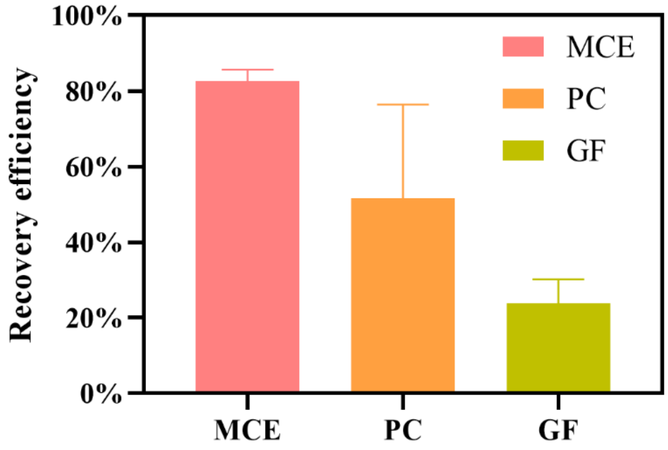

Figure S10 Recovery efficiency of microplastics intercepted by three kinds of filter membranes. The suspension of 10–40 μm PS standard microspheres was filtered through three kinds of filter membranes (pore size of 1.0 μm), including mixed cellulose esters (MCE) membrane, polycarbonate (PC) membrane, and glass fiber (GF) membrane, which was suspended with 15% DMSO for elution. The PS standard microspheres of elution samples were counted under the microscope.

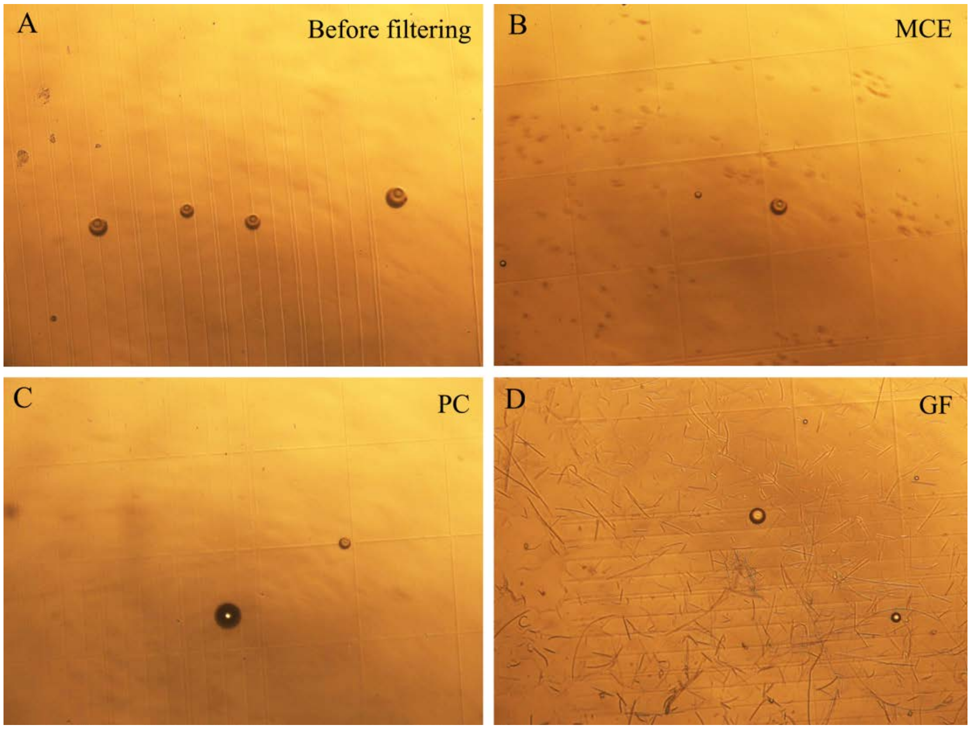

Figure S11 Microscopic image of microplastic suspension intercepted by three kinds of filter membranes. The suspension of 10–40 μm PS standard microspheres was filtered through three kinds of filter membranes (pore size of 1.0 μm) and then was suspended with 15% DMSO for elution. The elution samples of PS standard microspheres were observed under the microscope. In Figure S10, A is the microplastic suspension before filtering, B is the microplastic suspension intercepted by 1.0 μm mixed cellulose esters (MCE) membrane, C is the microplastic suspension intercepted by 1.0 μm polycarbonate (PC) membrane, and D is the microplastic suspension intercepted by 1.0 μm glass fiber (GF) membrane.

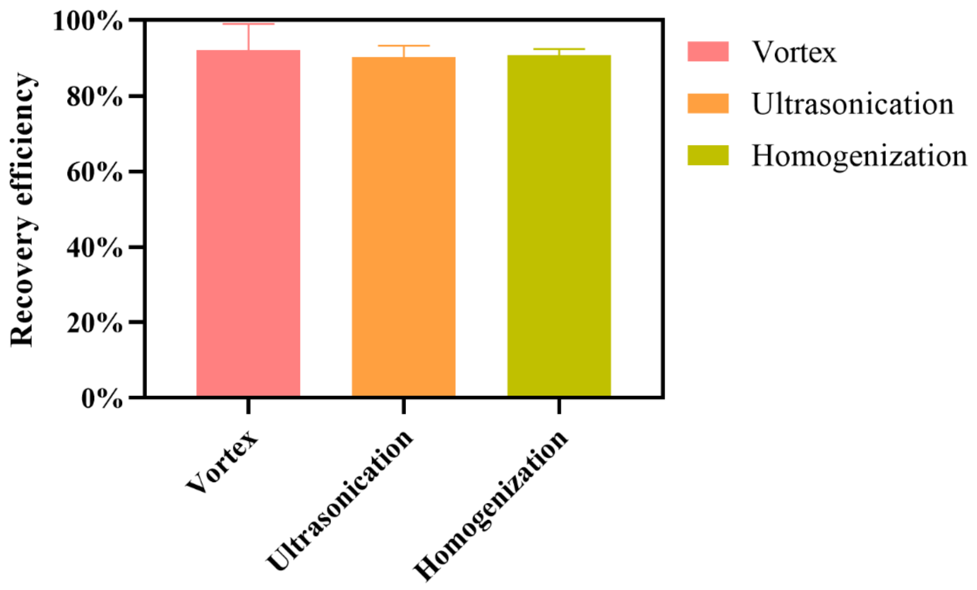

Figure S12 Recovery efficiency of three kinds of elution methods for microplastics intercepted by the filter membrane. The suspension of 10 μm–40 μm PS standard microspheres was filtered through a 1.0 μm mixed cellulose esters (MCE) membrane and then was eluted with 15% DMSO by three different elution methods, including vortex, ultrasonication, and homogenization. The PS standard microspheres of elution samples were counted under the microscope.

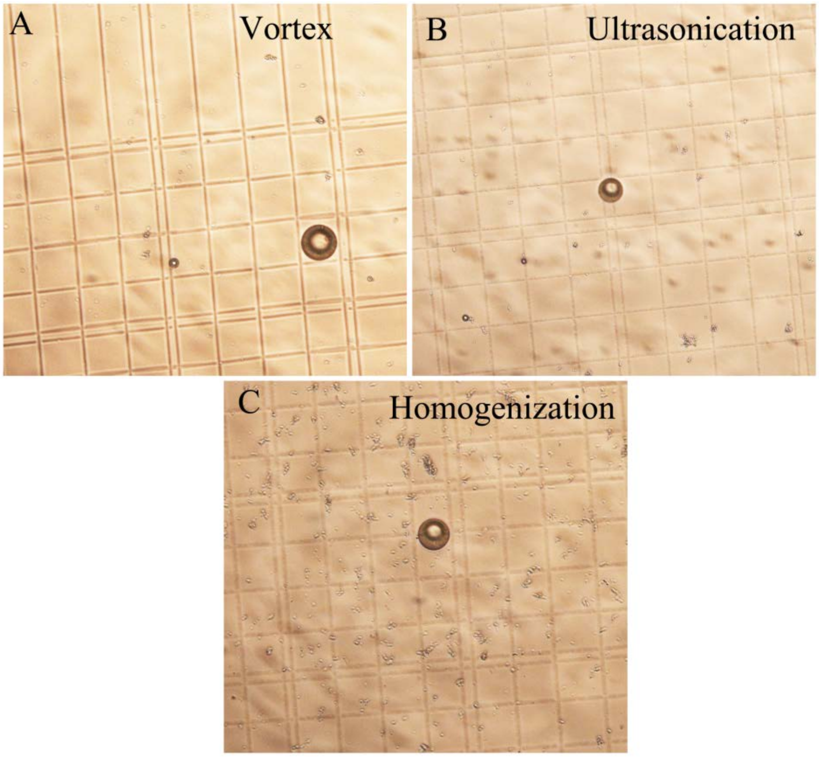

Figure S13 Microscopic images of the microplastic suspension eluted by three kinds of elution methods. The suspension of 10–40 μm PS standard microspheres was filtered through 1.0 μm mixed cellulose esters (MCE) membrane and then was eluted with 15% DMSO by three different elution methods, including vortex, ultrasonication, and homogenization. The PS standard microspheres of elution samples were observed under the microscope. In Figure S12, A is the microplastic suspension eluted by vortex, B is the microplastic suspension eluted by ultrasonication, and C is the microplastic suspension eluted by homogenization.

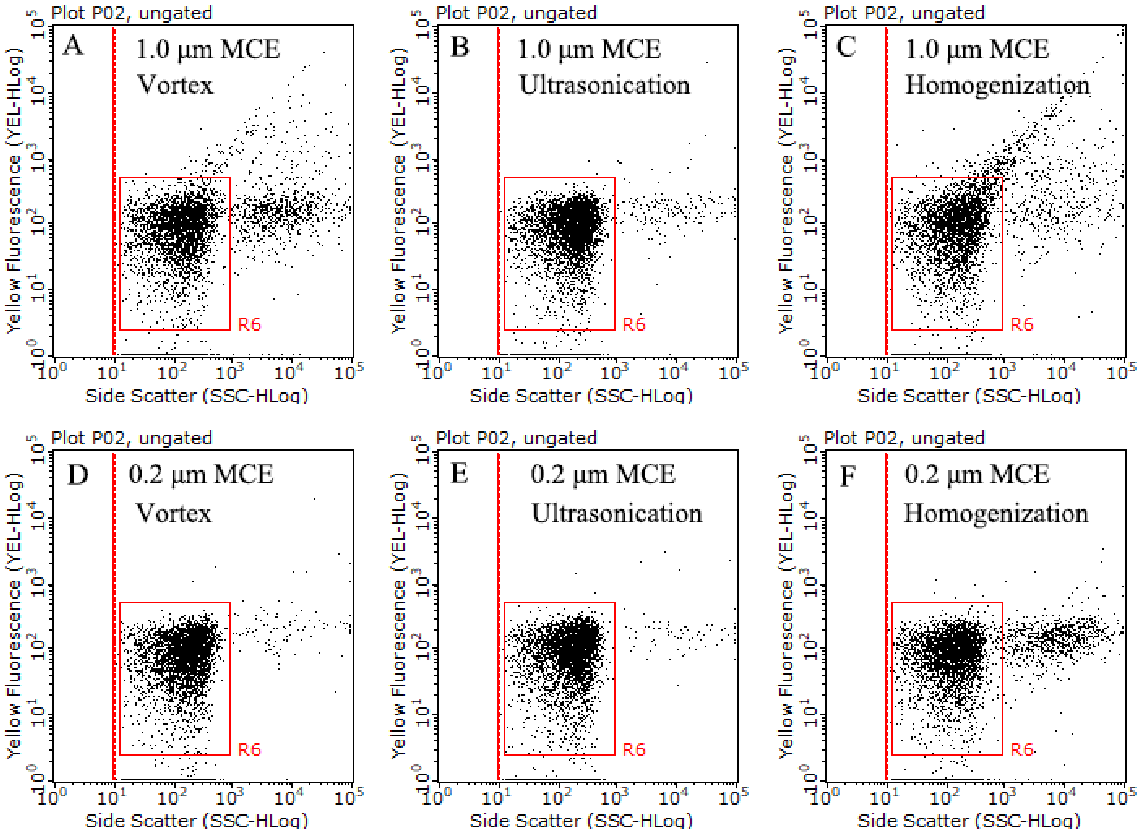

Figure S14 The damage to the MCE filter membranes by three different elution methods was compared. 50mL ultrapure water was sieved through 1.0 μm or 0.2 μm MCE filter membranes, then the filter membranes were cut into pieces, placed in glass tubes with 1mL of 30% DMSO, and treated with three different elution methods, including vortex, ultrasonication, and homogenization. After the elution suspension was sieved through 50 μm stainless steel mesh, then was stained with 15 μg/mL NR at room temperature for 10 min, and then the resuspension was analyzed by flow cytometry.

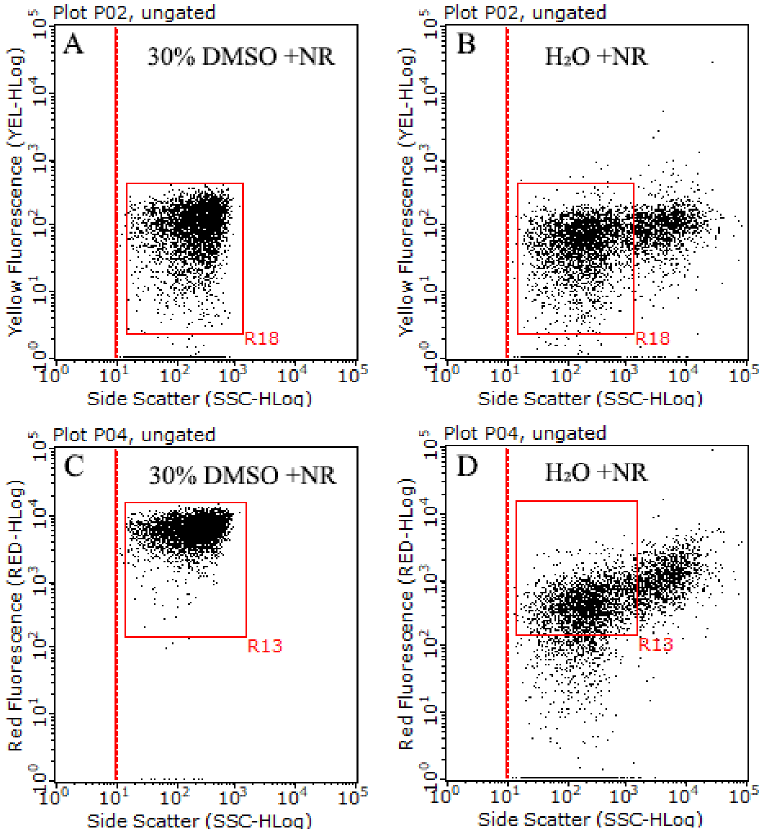

Figure S15 Dot plots of side scatter versus yellow fluorescence and side scatter versus red fluorescence for 15 μg/mL NR in 20% DMSO(A, C) or H_2O (B, D).
